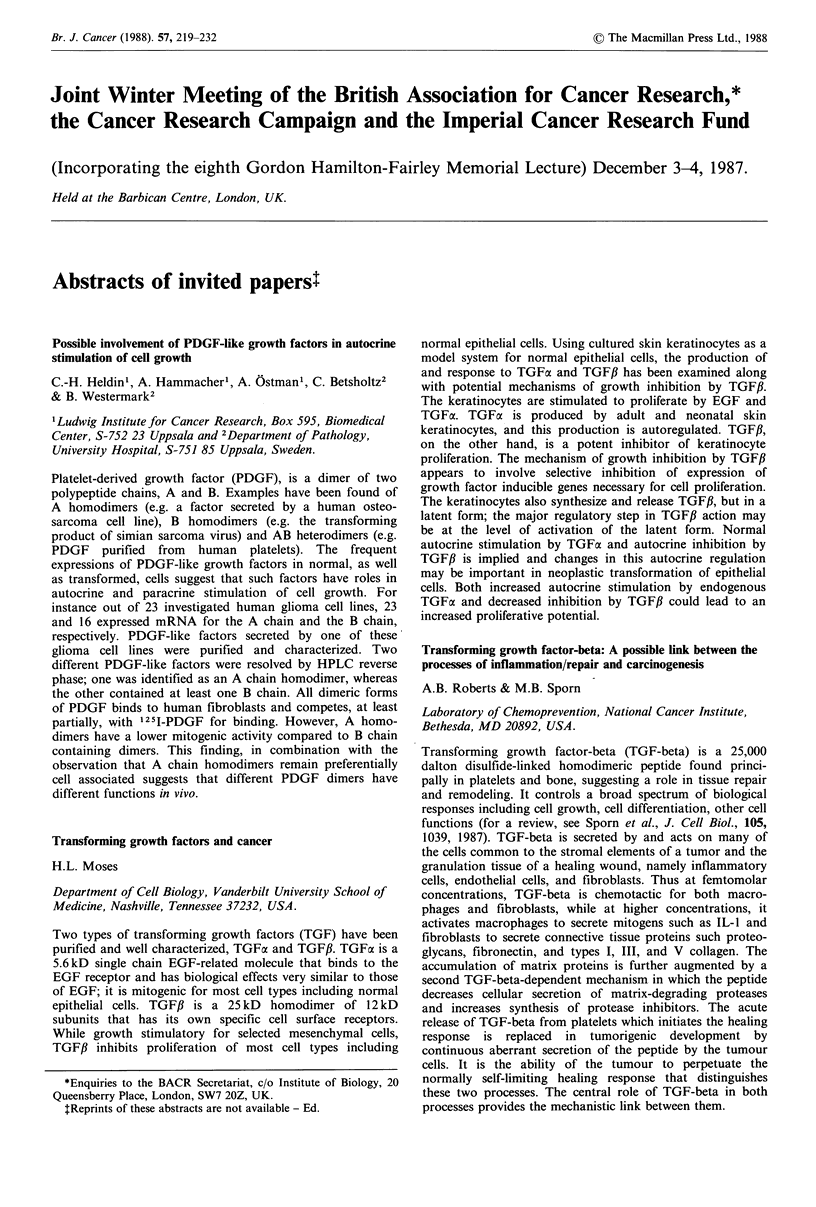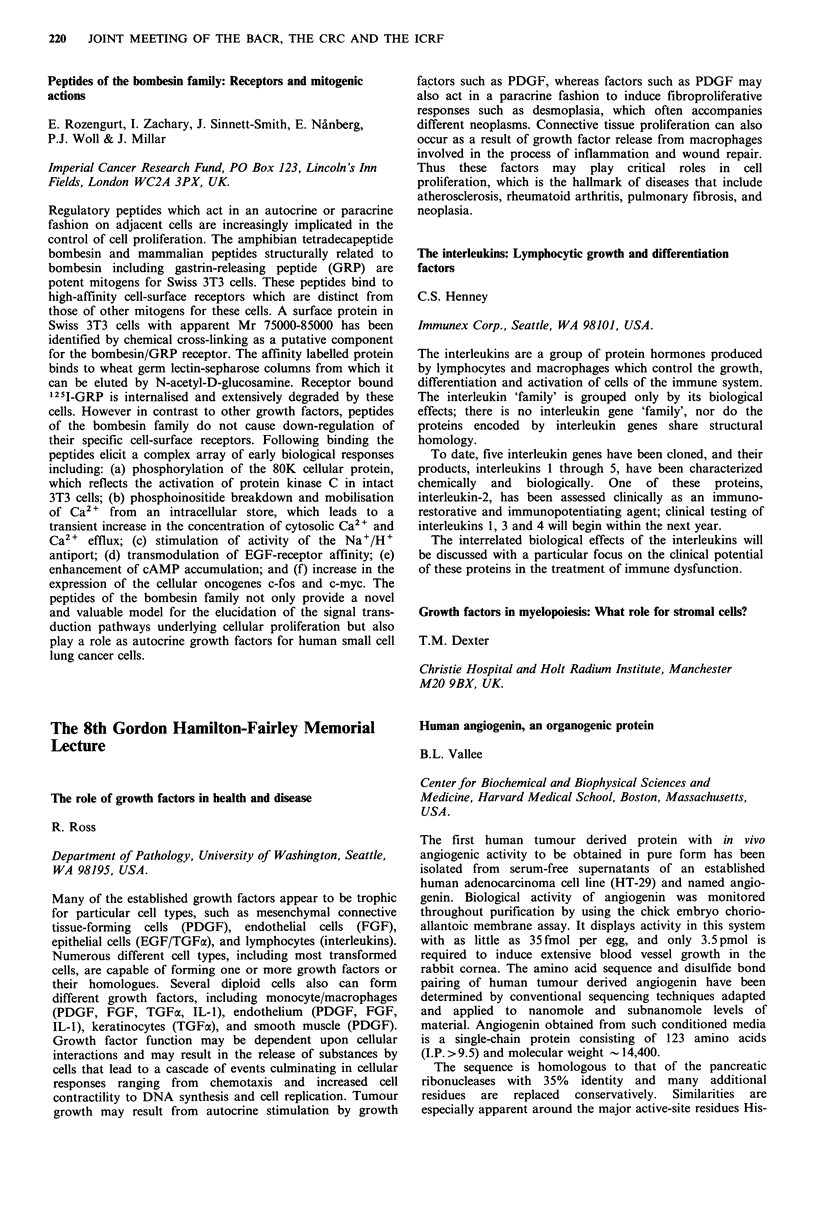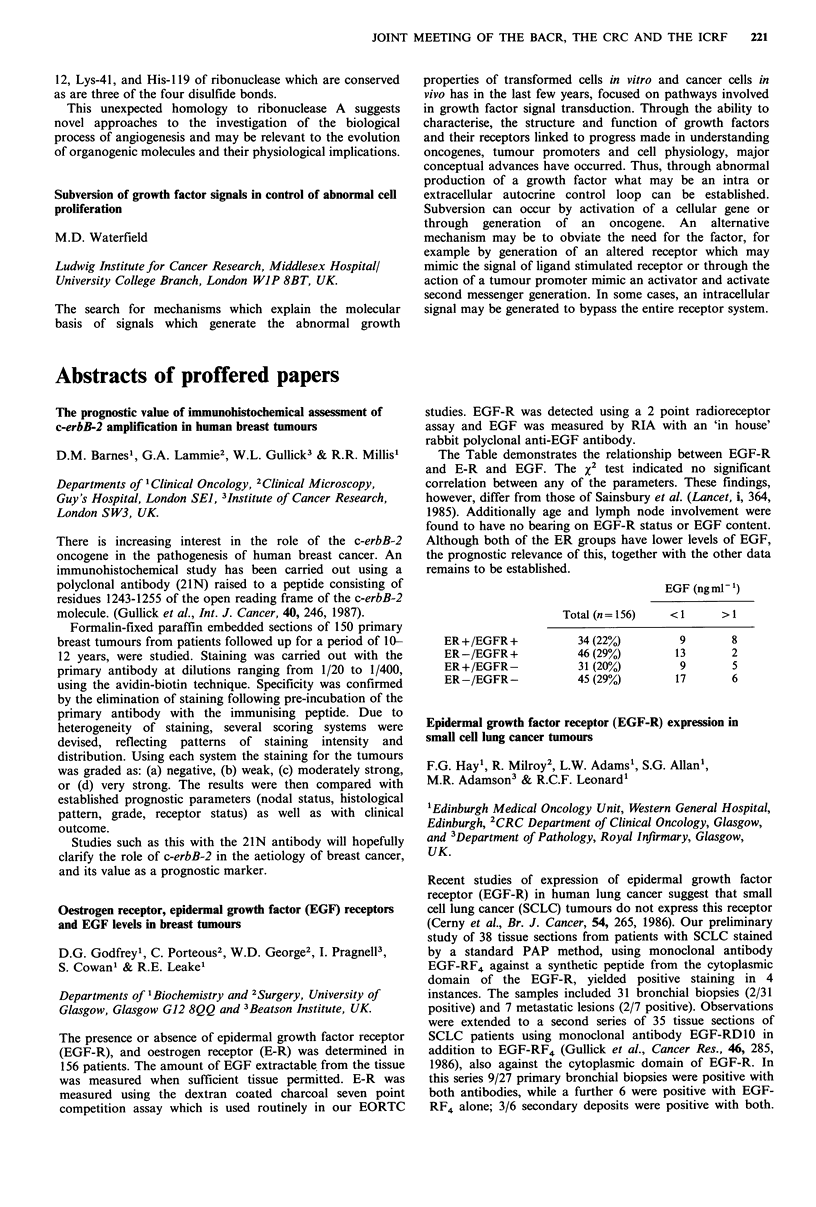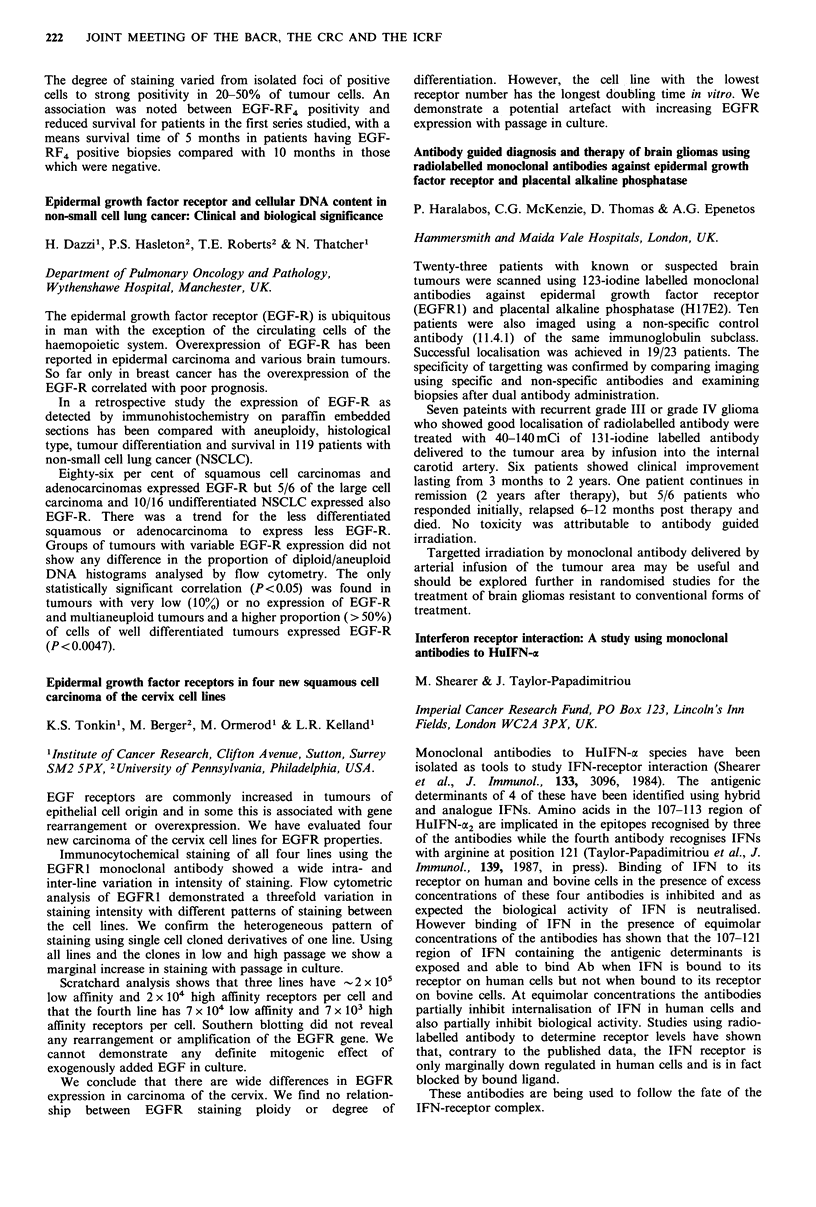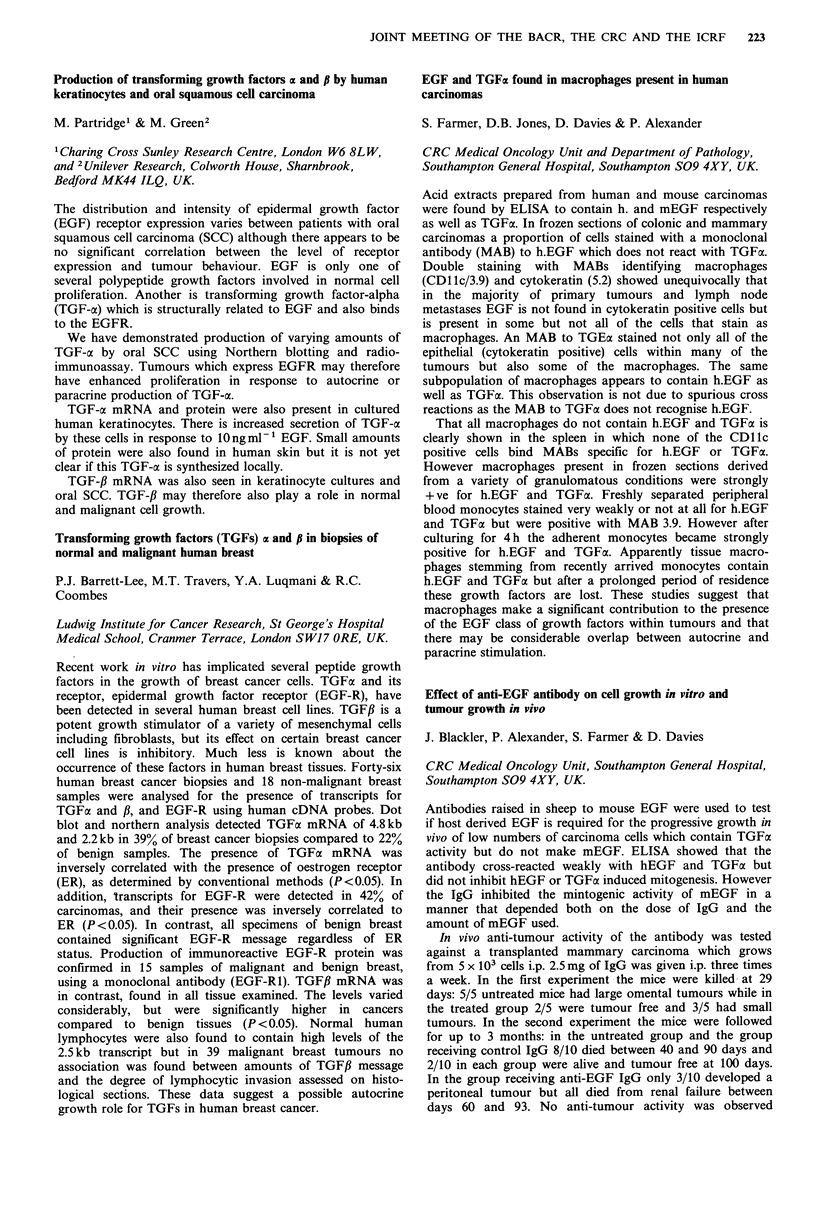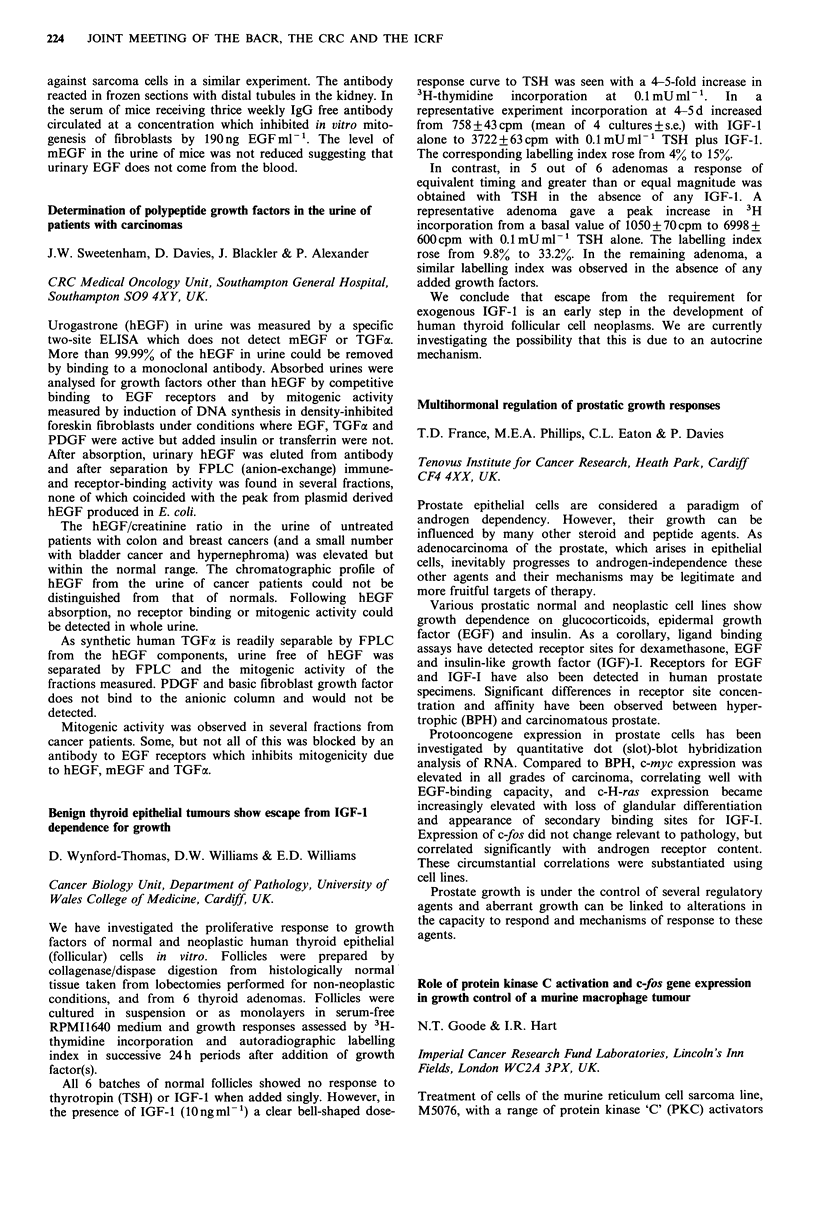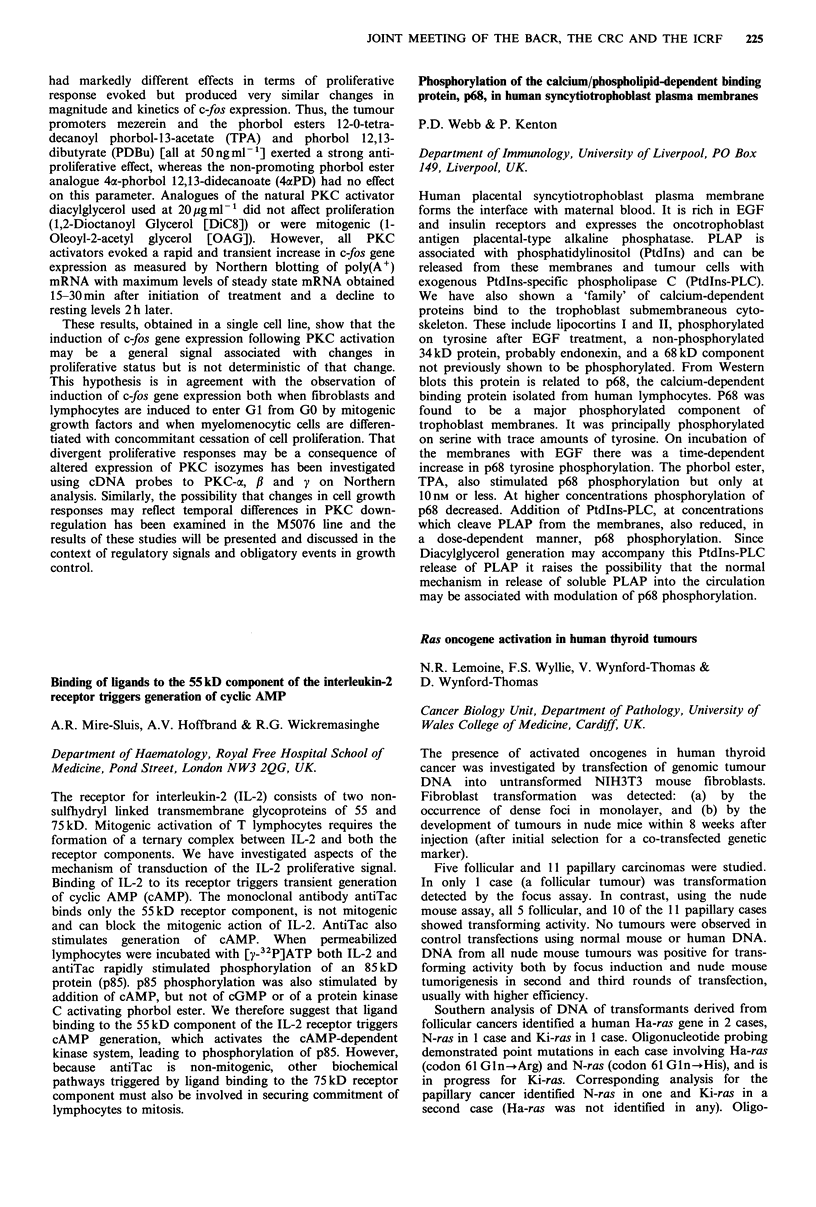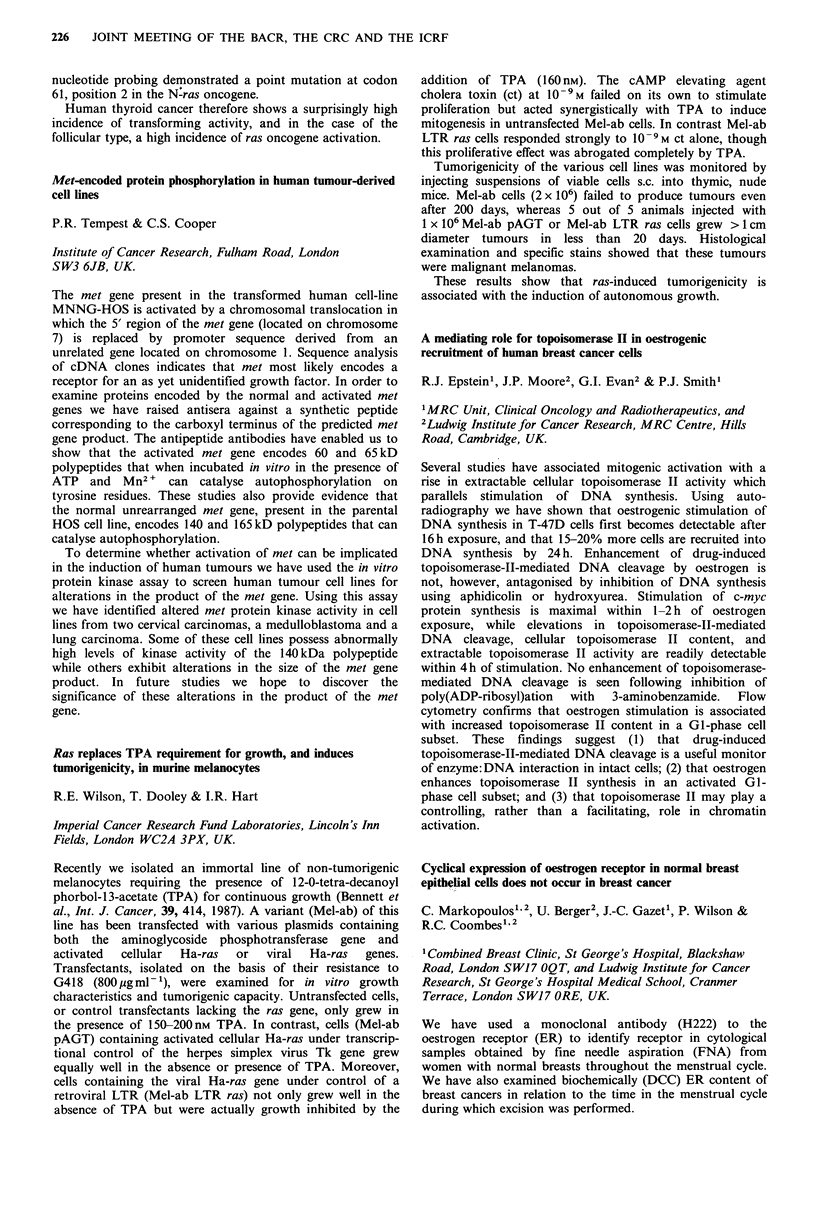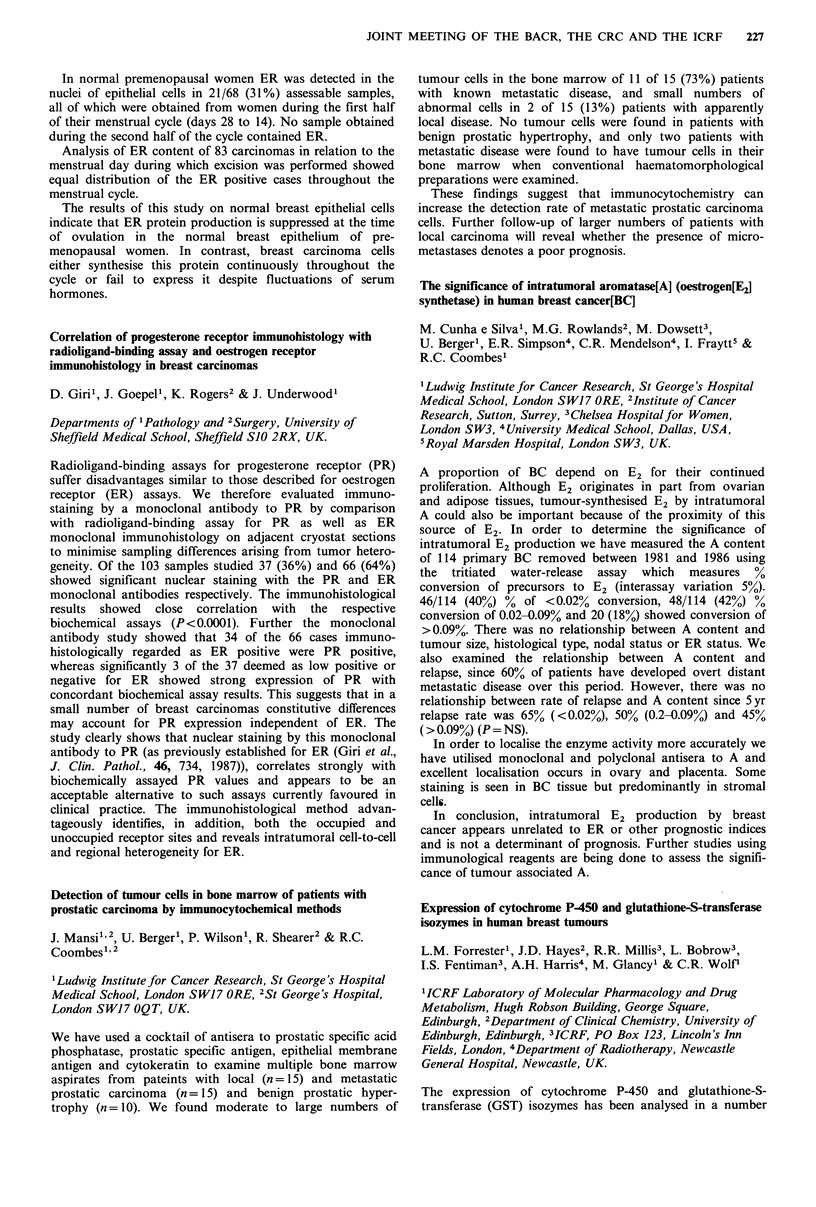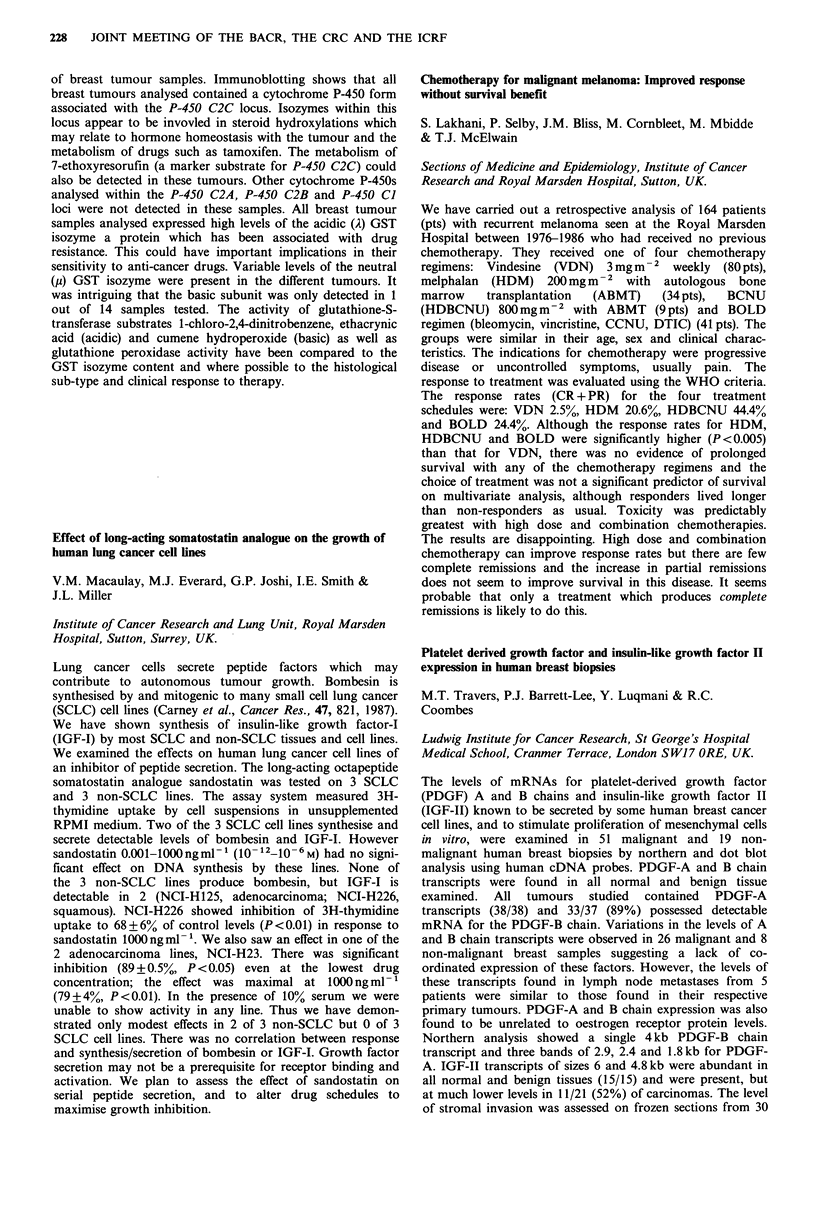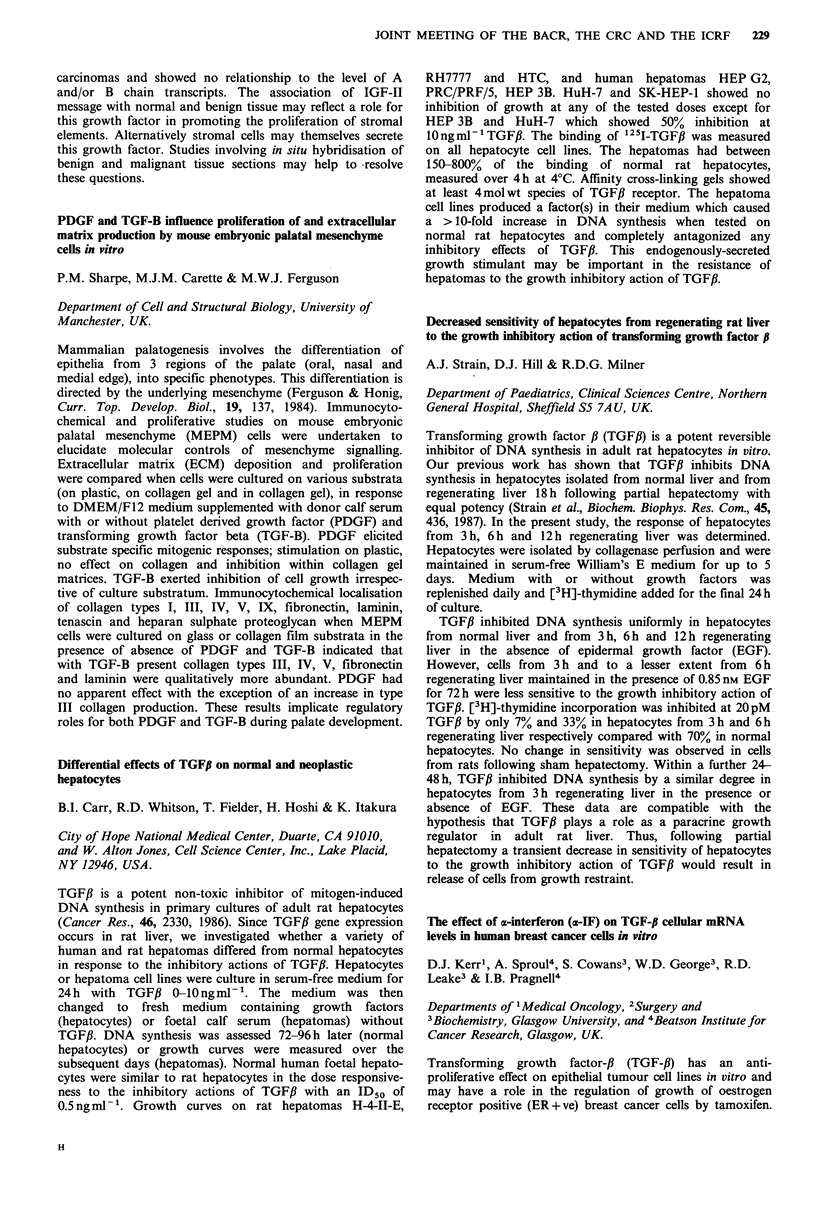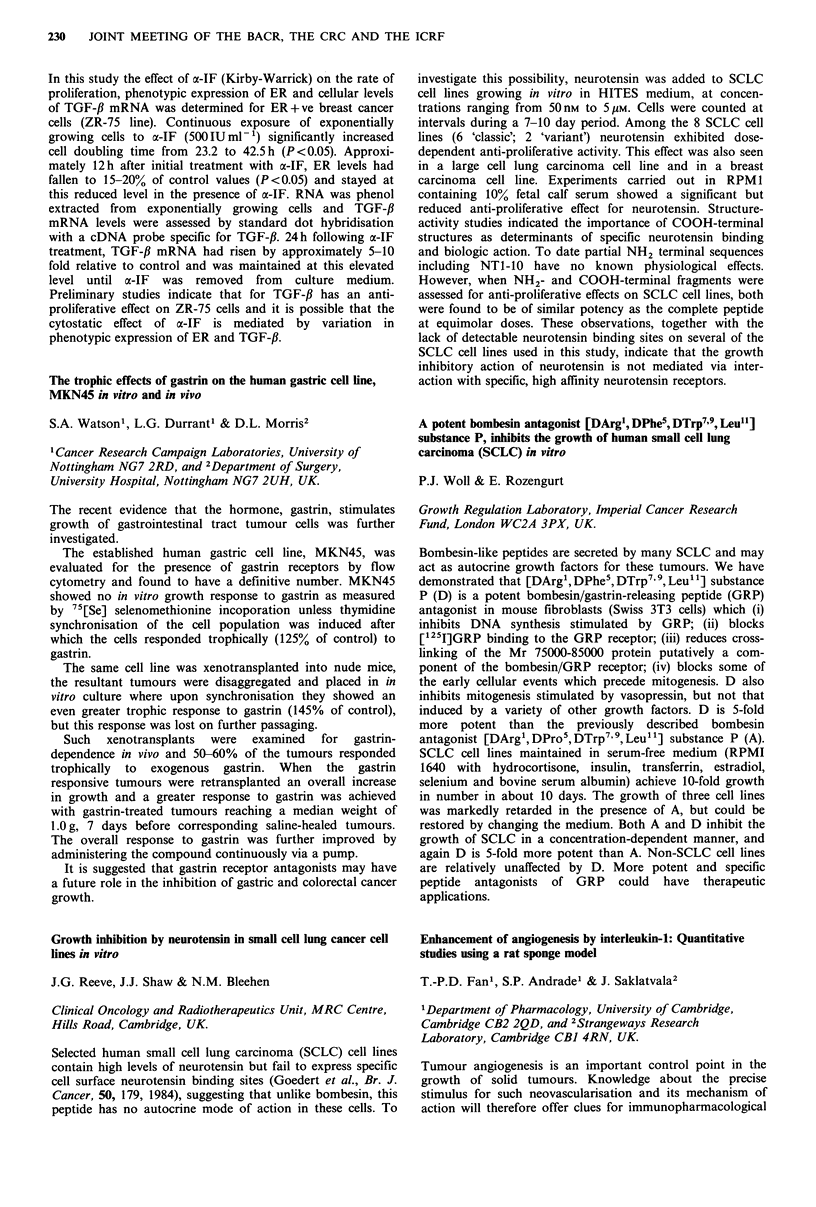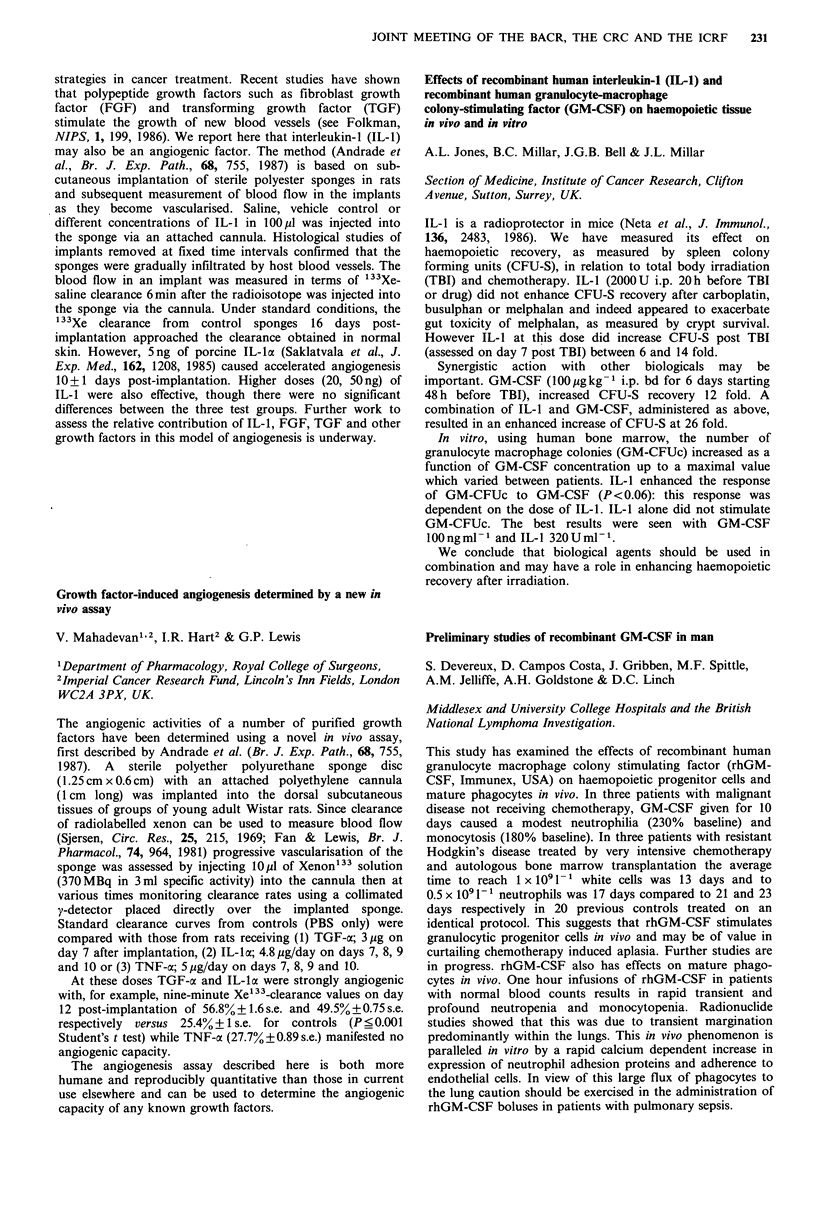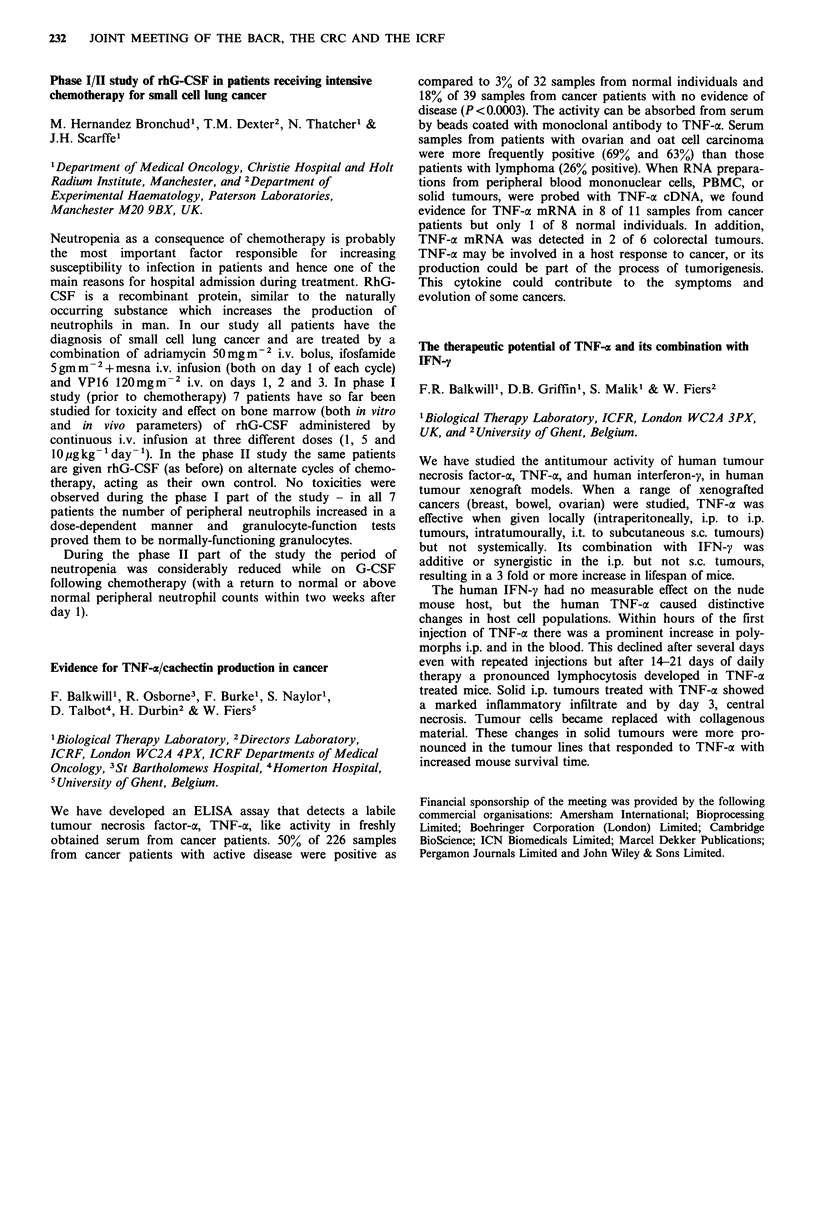# Joint winter meeting of the British Association for Cancer Research, the Cancer Research Campaign and the Imperial Cancer Research Fund. London, December 3-4, 1987. Abstracts.

**DOI:** 10.1038/bjc.1988.47

**Published:** 1988-02

**Authors:** 


*Br. J. Cancer* (1988). **57**, 219–232 © The Macmillan Press Ltd., 1988

### Joint Winter Meeting of the British Association for Cancer Research,* the Cancer Research Campaign and the Imperial Cancer Research Fund

(Incorporating the eighth Gordon Hamilton-Fairley Memorial Lecture) December 3–4, 1987.

*Held at the Barbican Centre, London, UK.*

### Abstracts of invited papers‡

### Possible involvement of PDGF-like growth factors in autocrine stimulation of cell growth

C.-H. Heldin[1], A. Hammacher[1], A. Östman[1], C. Betsholtz[2] & B. Westermark[2]

[1]*Ludwig Institute for Cancer Research, Box 595, Biomedical Center, S-752 23 Uppsala and* [2]*Department of Pathology, University Hospital, S-751 85 Uppsala, Sweden.*

Platelet-derived growth factor (PDGF), is a dimer of two polypeptide chains, A and B. Examples have been found of A homodimers (e.g. a factor secreted by a human osteosarcoma cell line), B homodimers (e.g. the transforming product of simian sarcoma virus) and AB heterodimers (e.g. PDGF purified from human platelets). The frequent expressions of PDGF-like growth factors in normal, as well as transformed, cells suggest that such factors have roles in autocrine and paracrine stimulation of cell growth. For instance out of 23 investigated human glioma cell lines, 23 and 16 expressed mRNA for the A chain and the B chain, respectively. PDGF-like factors secreted by one of these glioma cell lines were purified and characterized. Two different PDGF-like factors were resolved by HPLC reverse phase; one was identified as an A chain homodimer, whereas the other contained at least one B chain. All dimeric forms of PDGF binds to human fibroblasts and competes, at least partially, with $^{125}$I-PDGF for binding. However, A homodimers have a lower mitogenic activity compared to B chain containing dimers. This finding, in combination with the observation that A chain homodimers remain preferentially cell associated suggests that different PDGF dimers have different functions *in vivo*.

### Transforming growth factors and cancer

H.L. Moses

*Department of Cell Biology, Vanderbilt University School of Medicine, Nashville, Tennessee 37232, USA.*

Two types of transforming growth factors (TGF) have been purified and well characterized, TGFα and TGFβ. TGFα is a 5.6 kD single chain EGF-related molecule that binds to the EGF receptor and has biological effects very similar to those of EGF; it is mitogenic for most cell types including normal epithelial cells. TGFβ is a 25 kD homodimer of 12 kD subunits that has its own specific cell surface receptors. While growth stimulatory for selected mesenchymal cells, TGFβ inhibits proliferation of most cell types including normal epithelial cells. Using cultured skin keratinocytes as a model system for normal epithelial cells, the production of and response to TGFα and TGFβ has been examined along with potential mechanisms of growth inhibition by TGFβ. The keratinocytes are stimulated to proliferate by EGF and TGFα. TGFα is produced by adult and neonatal skin keratinocytes, and this production is autoregulated. TGFβ, on the other hand, is a potent inhibitor of keratinocyte proliferation. The mechanism of growth inhibition by TGFβ appears to involve selective inhibition of expression of growth factor inducible genes necessary for cell proliferation. The keratinocytes also synthesize and release TGFβ, but in a latent form; the major regulatory step in TGFβ action may be at the level of activation of the latent form. Normal autocrine stimulation by TGFα and autocrine inhibition by TGFβ is implied and changes in this autocrine regulation may be important in neoplastic transformation of epithelial cells. Both increased autocrine stimulation by endogenous TGFα and decreased inhibition by TGFβ could lead to an increased proliferative potential.

### Transforming growth factor-beta: A possible link between the processes of inflammation/repair and carcinogenesis

A.B. Roberts & M.B. Sporn

*Laboratory of Chemoprevention, National Cancer Institute, Bethesda, MD 20892, USA.*

Transforming growth factor-beta (TGF-beta) is a 25,000 dalton disulfide-linked homodimeric peptide found principally in platelets and bone, suggesting a role in tissue repair and remodeling. It controls a broad spectrum of biological responses including cell growth, cell differentiation, other cell functions (for a review, see Sporn *et al.*, *J. Cell Biol.*, **105,** 1039, 1987). TGF-beta is secreted by and acts on many of the cells common to the stromal elements of a tumor and the granulation tissue of a healing wound, namely inflammatory cells, endothelial cells, and fibroblasts. Thus at femtomolar concentrations, TGF-beta is chemotactic for both macrophages and fibroblasts, while at higher concentrations, it activates macrophages to secrete mitogens such as IL-1 and fibroblasts to secrete connective tissue proteins such proteoglycans, fibronectin, and types I, III, and V collagen. The accumulation of matrix proteins is further augmented by a second TGF-beta-dependent mechanism in which the peptide decreases cellular secretion of matrix-degrading proteases and increases synthesis of protease inhibitors. The acute release of TGF-beta from platelets which initiates the healing response is replaced in tumorigenic development by continuous aberrant secretion of the peptide by the tumour cells. It is the ability of the tumour to perpetuate the normally self-limiting healing response that distinguishes these two processes. The central role of TGF-beta in both processes provides the mechanistic link between them.

*Enquiries to the BACR Secretariat, c/o Institute of Biology, 20 Queensberry Place, London, SW7 20Z, UK.
‡Reprints of these abstracts are not available – Ed.

### Peptides of the bombesin family: Receptors and mitogenic actions

E. Rozengurt, I. Zachary, J. Sinnett-Smith, E. Nånberg, P.J. Woll & J. Millar

*Imperial Cancer Research Fund, PO Box 123, Lincoln's Inn Fields, London WC2A 3PX, UK.*

Regulatory peptides which act in an autocrine or paracrine fashion on adjacent cells are increasingly implicated in the control of cell proliferation. The amphibian tetradecapeptide bombesin and mammalian peptides structurally related to bombesin including gastrin-releasing peptide (GRP) are potent mitogens for Swiss 3T3 cells. These peptides bind to high-affinity cell-surface receptors which are distinct from those of other mitogens for these cells. A surface protein in Swiss 3T3 cells with apparent Mr 75000-85000 has been identified by chemical cross-linking as a putative component for the bombesin/GRP receptor. The affinity labelled protein binds to wheat germ lectin-sepharose columns from which it can be eluted by N-acetyl-D-glucosamine. Receptor bound $^{125}$I-GRP is internalised and extensively degraded by these cells. However in contrast to other growth factors, peptides of the bombesin family do not cause down-regulation of their specific cell-surface receptors. Following binding the peptides elicit a complex array of early biological responses including: (a) phosphorylation of the 80K cellular protein, which reflects the activation of protein kinase C in intact 3T3 cells; (b) phosphoinositide breakdown and mobilisation of $Ca^{2+}$ from an intracellular store, which leads to a transient increase in the concentration of cytosolic $Ca^{2+}$ and $Ca^{2+}$ efflux; (c) stimulation of activity of the $Na^+/H^+$ antiport; (d) transmodulation of EGF-receptor affinity; (e) enhancement of cAMP accumulation; and (f) increase in the expression of the cellular oncogenes c-fos and c-myc. The peptides of the bombesin family not only provide a novel and valuable model for the elucidation of the signal transduction pathways underlying cellular proliferation but also play a role as autocrine growth factors for human small cell lung cancer cells.

### The 8th Gordon Hamilton-Fairley Memorial Lecture

### The role of growth factors in health and disease

R. Ross

*Department of Pathology, University of Washington, Seattle, WA 98195, USA.*

Many of the established growth factors appear to be trophic for particular cell types, such as mesenchymal connective tissue-forming cells (PDGF), endothelial cells (FGF), epithelial cells (EGF/TGFα), and lymphocytes (interleukins). Numerous different cell types, including most transformed cells, are capable of forming one or more growth factors or their homologues. Several diploid cells also can form different growth factors, including monocyte/macrophages (PDGF, FGF, TGFα, IL-1), endothelium (PDGF, FGF, IL-1), keratinocytes (TGFα), and smooth muscle (PDGF). Growth factor function may be dependent upon cellular interactions and may result in the release of substances by cells that lead to a cascade of events culminating in cellular responses ranging from chemotaxis and increased cell contractility to DNA synthesis and cell replication. Tumour growth may result from autocrine stimulation by growth factors such as PDGF, whereas factors such as PDGF may also act in a paracrine fashion to induce fibroproliferative responses such as desmoplasia, which often accompanies different neoplasms. Connective tissue proliferation can also occur as a result of growth factor release from macrophages involved in the process of inflammation and wound repair. Thus these factors may play critical roles in cell proliferation, which is the hallmark of diseases that include atherosclerosis, rheumatoid arthritis, pulmonary fibrosis, and neoplasia.

### The interleukins: Lymphocytic growth and differentiation factors

C.S. Henney

*Immunex Corp., Seattle, WA 98101, USA.*

The interleukins are a group of protein hormones produced by lymphocytes and macrophages which control the growth, differentiation and activation of cells of the immune system. The interleukin 'family' is grouped only by its biological effects; there is no interleukin gene 'family', nor do the proteins encoded by interleukin genes share structural homology.

To date, five interleukin genes have been cloned, and their products, interleukins 1 through 5, have been characterized chemically and biologically. One of these proteins, interleukin-2, has been assessed clinically as an immunorestorative and immunopotentiating agent; clinical testing of interleukins 1, 3 and 4 will begin within the next year.

The interrelated biological effects of the interleukins will be discussed with a particular focus on the clinical potential of these proteins in the treatment of immune dysfunction.

### Growth factors in myelopoiesis: What role for stromal cells?

T.M. Dexter

*Christie Hospital and Holt Radium Institute, Manchester M20 9BX, UK.*

### Human angiogenin, an organogenic protein

B.L. Vallee

*Center for Biochemical and Biophysical Sciences and Medicine, Harvard Medical School, Boston, Massachusetts, USA.*

The first human tumour derived protein with *in vivo* angiogenic activity to be obtained in pure form has been isolated from serum-free supernatants of an established human adenocarcinoma cell line (HT-29) and named angiogenin. Biological activity of angiogenin was monitored throughout purification by using the chick embryo chorioallantoic membrane assay. It displays activity in this system with as little as 35 fmol per egg, and only 3.5 pmol is required to induce extensive blood vessel growth in the rabbit cornea. The amino acid sequence and disulfide bond pairing of human tumour derived angiogenin have been determined by conventional sequencing techniques adapted and applied to nanomole and subnanomole levels of material. Angiogenin obtained from such conditioned media is a single-chain protein consisting of 123 amino acids (I.P. $>9.5$) and molecular weight $\sim 14{,}400$.

The sequence is homologous to that of the pancreatic ribonucleases with 35% identity and many additional residues are replaced conservatively. Similarities are especially apparent around the major active-site residues His-

12, Lys-41, and His-119 of ribonuclease which are conserved as are three of the four disulfide bonds.

This unexpected homology to ribonuclease A suggests novel approaches to the investigation of the biological process of angiogenesis and may be relevant to the evolution of organogenic molecules and their physiological implications.

### Subversion of growth factor signals in control of abnormal cell proliferation

M.D. Waterfield

*Ludwig Institute for Cancer Research, Middlesex Hospital/ University College Branch, London W1P 8BT, UK.*

The search for mechanisms which explain the molecular basis of signals which generate the abnormal growth properties of transformed cells *in vitro* and cancer cells *in vivo* has in the last few years, focused on pathways involved in growth factor signal transduction. Through the ability to characterise, the structure and function of growth factors and their receptors linked to progress made in understanding oncogenes, tumour promoters and cell physiology, major conceptual advances have occurred. Thus, through abnormal production of a growth factor what may be an intra or extracellular autocrine control loop can be established. Subversion can occur by activation of a cellular gene or through generation of an oncogene. An alternative mechanism may be to obviate the need for the factor, for example by generation of an altered receptor which may mimic the signal of ligand stimulated receptor or through the action of a tumour promoter mimic an activator and activate second messenger generation. In some cases, an intracellular signal may be generated to bypass the entire receptor system.

### Abstracts of proffered papers

### The prognostic value of immunohistochemical assessment of c-*erbB-2* amplification in human breast tumours

D.M. Barnes[1], G.A. Lammie[2], W.L. Gullick[3] & R.R. Millis[1]

*Departments of [1]Clinical Oncology, [2]Clinical Microscopy, Guy's Hospital, London SE1, [3]Institute of Cancer Research, London SW3, UK.*

There is increasing interest in the role of the c-*erbB-2* oncogene in the pathogenesis of human breast cancer. An immunohistochemical study has been carried out using a polyclonal antibody (21N) raised to a peptide consisting of residues 1243-1255 of the open reading frame of the c-*erbB-2* molecule. (Gullick *et al.*, *Int. J. Cancer*, **40**, 246, 1987).

Formalin-fixed paraffin embedded sections of 150 primary breast tumours from patients followed up for a period of 10–12 years, were studied. Staining was carried out with the primary antibody at dilutions ranging from 1/20 to 1/400, using the avidin-biotin technique. Specificity was confirmed by the elimination of staining following pre-incubation of the primary antibody with the immunising peptide. Due to heterogeneity of staining, several scoring systems were devised, reflecting patterns of staining intensity and distribution. Using each system the staining for the tumours was graded as: (a) negative, (b) weak, (c) moderately strong, or (d) very strong. The results were then compared with established prognostic parameters (nodal status, histological pattern, grade, receptor status) as well as with clinical outcome.

Studies such as this with the 21N antibody will hopefully clarify the role of c-*erbB-2* in the aetiology of breast cancer, and its value as a prognostic marker.

### Oestrogen receptor, epidermal growth factor (EGF) receptors and EGF levels in breast tumours

D.G. Godfrey[1], C. Porteous[2], W.D. George[2], I. Pragnell[3], S. Cowan[1] & R.E. Leake[1]

*Departments of [1]Biochemistry and [2]Surgery, University of Glasgow, Glasgow G12 8QQ and [3]Beatson Institute, UK.*

The presence or absence of epidermal growth factor receptor (EGF-R), and oestrogen receptor (E-R) was determined in 156 patients. The amount of EGF extractable from the tissue was measured when sufficient tissue permitted. E-R was measured using the dextran coated charcoal seven point competition assay which is used routinely in our EORTC studies. EGF-R was detected using a 2 point radioreceptor assay and EGF was measured by RIA with an 'in house' rabbit polyclonal anti-EGF antibody.

The Table demonstrates the relationship between EGF-R and E-R and EGF. The $\chi^2$ test indicated no significant correlation between any of the parameters. These findings, however, differ from those of Sainsbury *et al.* (*Lancet*, **i**, 364, 1985). Additionally age and lymph node involvement were found to have no bearing on EGF-R status or EGF content. Although both of the ER groups have lower levels of EGF, the prognostic relevance of this, together with the other data remains to be established.

| | | EGF ($ng\,ml^{-1}$) | |
|---|---|---|---|
| | Total ($n=156$) | <1 | >1 |
| ER+/EGFR+ | 34 (22%) | 9 | 8 |
| ER−/EGFR+ | 46 (29%) | 13 | 2 |
| ER+/EGFR− | 31 (20%) | 9 | 5 |
| ER−/EGFR− | 45 (29%) | 17 | 6 |

### Epidermal growth factor receptor (EGF-R) expression in small cell lung cancer tumours

F.G. Hay[1], R. Milroy[2], L.W. Adams[1], S.G. Allan[1], M.R. Adamson[3] & R.C.F. Leonard[1]

*[1]Edinburgh Medical Oncology Unit, Western General Hospital, Edinburgh, [2]CRC Department of Clinical Oncology, Glasgow, and [3]Department of Pathology, Royal Infirmary, Glasgow, UK.*

Recent studies of expression of epidermal growth factor receptor (EGF-R) in human lung cancer suggest that small cell lung cancer (SCLC) tumours do not express this receptor (Cerny *et al.*, *Br. J. Cancer*, **54**, 265, 1986). Our preliminary study of 38 tissue sections from patients with SCLC stained by a standard PAP method, using monoclonal antibody EGF-$RF_4$ against a synthetic peptide from the cytoplasmic domain of the EGF-R, yielded positive staining in 4 instances. The samples included 31 bronchial biopsies (2/31 positive) and 7 metastatic lesions (2/7 positive). Observations were extended to a second series of 35 tissue sections of SCLC patients using monoclonal antibody EGF-RD10 in addition to EGF-$RF_4$ (Gullick *et al.*, *Cancer Res.*, **46**, 285, 1986), also against the cytoplasmic domain of EGF-R. In this series 9/27 primary bronchial biopsies were positive with both antibodies, while a further 6 were positive with EGF-$RF_4$ alone; 3/6 secondary deposits were positive with both.

The degree of staining varied from isolated foci of positive cells to strong positivity in 20–50% of tumour cells. An association was noted between EGF-$RF_4$ positivity and reduced survival for patients in the first series studied, with a means survival time of 5 months in patients having EGF-$RF_4$ positive biopsies compared with 10 months in those which were negative.

### Epidermal growth factor receptor and cellular DNA content in non-small cell lung cancer: Clinical and biological significance

H. Dazzi[1], P.S. Hasleton[2], T.E. Roberts[2] & N. Thatcher[1]

*Department of Pulmonary Oncology and Pathology, Wythenshawe Hospital, Manchester, UK.*

The epidermal growth factor receptor (EGF-R) is ubiquitous in man with the exception of the circulating cells of the haemopoietic system. Overexpression of EGF-R has been reported in epidermal carcinoma and various brain tumours. So far only in breast cancer has the overexpression of the EGF-R correlated with poor prognosis.

In a retrospective study the expression of EGF-R as detected by immunohistochemistry on paraffin embedded sections has been compared with aneuploidy, histological type, tumour differentiation and survival in 119 patients with non-small cell lung cancer (NSCLC).

Eighty-six per cent of squamous cell carcinomas and adenocarcinomas expressed EGF-R but 5/6 of the large cell carcinoma and 10/16 undifferentiated NSCLC expressed also EGF-R. There was a trend for the less differentiated squamous or adenocarcinoma to express less EGF-R. Groups of tumours with variable EGF-R expression did not show any difference in the proportion of diploid/aneuploid DNA histograms analysed by flow cytometry. The only statistically significant correlation ($P<0.05$) was found in tumours with very low (10%) or no expression of EGF-R and multianeuploid tumours and a higher proportion (>50%) of cells of well differentiated tumours expressed EGF-R ($P<0.0047$).

### Epidermal growth factor receptors in four new squamous cell carcinoma of the cervix cell lines

K.S. Tonkin[1], M. Berger[2], M. Ormerod[1] & L.R. Kelland[1]

[1]*Institute of Cancer Research, Clifton Avenue, Sutton, Surrey SM2 5PX,* [2]*University of Pennsylvania, Philadelphia, USA.*

EGF receptors are commonly increased in tumours of epithelial cell origin and in some this is associated with gene rearrangement or overexpression. We have evaluated four new carcinoma of the cervix cell lines for EGFR properties.

Immunocytochemical staining of all four lines using the EGFR1 monoclonal antibody showed a wide intra- and inter-line variation in intensity of staining. Flow cytometric analysis of EGFR1 demonstrated a threefold variation in staining intensity with different patterns of staining between the cell lines. We confirm the heterogeneous pattern of staining using single cell cloned derivatives of one line. Using all lines and the clones in low and high passage we show a marginal increase in staining with passage in culture.

Scratchard analysis shows that three lines have $\sim 2\times10^5$ low affinity and $2\times10^4$ high affinity receptors per cell and that the fourth line has $7\times10^4$ low affinity and $7\times10^3$ high affinity receptors per cell. Southern blotting did not reveal any rearrangement or amplification of the EGFR gene. We cannot demonstrate any definite mitogenic effect of exogenously added EGF in culture.

We conclude that there are wide differences in EGFR expression in carcinoma of the cervix. We find no relationship between EGFR staining ploidy or degree of differentiation. However, the cell line with the lowest receptor number has the longest doubling time *in vitro*. We demonstrate a potential artefact with increasing EGFR expression with passage in culture.

### Antibody guided diagnosis and therapy of brain gliomas using radiolabelled monoclonal antibodies against epidermal growth factor receptor and placental alkaline phosphatase

P. Haralabos, C.G. McKenzie, D. Thomas & A.G. Epenetos

*Hammersmith and Maida Vale Hospitals, London, UK.*

Twenty-three patients with known or suspected brain tumours were scanned using 123-iodine labelled monoclonal antibodies against epidermal growth factor receptor (EGFR1) and placental alkaline phosphatase (H17E2). Ten patients were also imaged using a non-specific control antibody (11.4.1) of the same immunoglobulin subclass. Successful localisation was achieved in 19/23 patients. The specificity of targetting was confirmed by comparing imaging using specific and non-specific antibodies and examining biopsies after dual antibody administration.

Seven pateints with recurrent grade III or grade IV glioma who showed good localisation of radiolabelled antibody were treated with 40–140 mCi of 131-iodine labelled antibody delivered to the tumour area by infusion into the internal carotid artery. Six patients showed clinical improvement lasting from 3 months to 2 years. One patient continues in remission (2 years after therapy), but 5/6 patients who responded initially, relapsed 6–12 months post therapy and died. No toxicity was attributable to antibody guided irradiation.

Targetted irradiation by monoclonal antibody delivered by arterial infusion of the tumour area may be useful and should be explored further in randomised studies for the treatment of brain gliomas resistant to conventional forms of treatment.

### Interferon receptor interaction: A study using monoclonal antibodies to HuIFN-α

M. Shearer & J. Taylor-Papadimitriou

*Imperial Cancer Research Fund, PO Box 123, Lincoln's Inn Fields, London WC2A 3PX, UK.*

Monoclonal antibodies to HuIFN-α species have been isolated as tools to study IFN-receptor interaction (Shearer *et al.*, *J. Immunol.*, **133**, 3096, 1984). The antigenic determinants of 4 of these have been identified using hybrid and analogue IFNs. Amino acids in the 107–113 region of HuIFN-$\alpha_2$ are implicated in the epitopes recognised by three of the antibodies while the fourth antibody recognises IFNs with arginine at position 121 (Taylor-Papadimitriou *et al.*, *J. Immunol.*, **139**, 1987, in press). Binding of IFN to its receptor on human and bovine cells in the presence of excess concentrations of these four antibodies is inhibited and as expected the biological activity of IFN is neutralised. However binding of IFN in the presence of equimolar concentrations of the antibodies has shown that the 107–121 region of IFN containing the antigenic determinants is exposed and able to bind Ab when IFN is bound to its receptor on human cells but not when bound to its receptor on bovine cells. At equimolar concentrations the antibodies partially inhibit internalisation of IFN in human cells and also partially inhibit biological activity. Studies using radio-labelled antibody to determine receptor levels have shown that, contrary to the published data, the IFN receptor is only marginally down regulated in human cells and is in fact blocked by bound ligand.

These antibodies are being used to follow the fate of the IFN-receptor complex.

**Production of transforming growth factors α and β by human keratinocytes and oral squamous cell carcinoma**

M. Partridge[1] & M. Green[2]

[1]*Charing Cross Sunley Research Centre, London W6 8LW, and* [2]*Unilever Research, Colworth House, Sharnbrook, Bedford MK44 ILQ, UK.*

The distribution and intensity of epidermal growth factor (EGF) receptor expression varies between patients with oral squamous cell carcinoma (SCC) although there appears to be no significant correlation between the level of receptor expression and tumour behaviour. EGF is only one of several polypeptide growth factors involved in normal cell proliferation. Another is transforming growth factor-alpha (TGF-α) which is structurally related to EGF and also binds to the EGFR.

We have demonstrated production of varying amounts of TGF-α by oral SCC using Northern blotting and radioimmunoassay. Tumours which express EGFR may therefore have enhanced proliferation in response to autocrine or paracrine production of TGF-α.

TGF-α mRNA and protein were also present in cultured human keratinocytes. There is increased secretion of TGF-α by these cells in response to $10\,ng\,ml^{-1}$ EGF. Small amounts of protein were also found in human skin but it is not yet clear if this TGF-α is synthesized locally.

TGF-β mRNA was also seen in keratinocyte cultures and oral SCC. TGF-β may therefore also play a role in normal and malignant cell growth.

**Transforming growth factors (TGFs) α and β in biopsies of normal and malignant human breast**

P.J. Barrett-Lee, M.T. Travers, Y.A. Luqmani & R.C. Coombes

*Ludwig Institute for Cancer Research, St George's Hospital Medical School, Cranmer Terrace, London SW17 0RE, UK.*

Recent work *in vitro* has implicated several peptide growth factors in the growth of breast cancer cells. TGFα and its receptor, epidermal growth factor receptor (EGF-R), have been detected in several human breast cell lines. TGFβ is a potent growth stimulator of a variety of mesenchymal cells including fibroblasts, but its effect on certain breast cancer cell lines is inhibitory. Much less is known about the occurrence of these factors in human breast tissues. Forty-six human breast cancer biopsies and 18 non-malignant breast samples were analysed for the presence of transcripts for TGFα and β, and EGF-R using human cDNA probes. Dot blot and northern analysis detected TGFα mRNA of 4.8 kb and 2.2 kb in 39% of breast cancer biopsies compared to 22% of benign samples. The presence of TGFα mRNA was inversely correlated with the presence of oestrogen receptor (ER), as determined by conventional methods ($P<0.05$). In addition, transcripts for EGF-R were detected in 42% of carcinomas, and their presence was inversely correlated to ER ($P<0.05$). In contrast, all specimens of benign breast contained significant EGF-R message regardless of ER status. Production of immunoreactive EGF-R protein was confirmed in 15 samples of malignant and benign breast, using a monoclonal antibody (EGF-R1). TGFβ mRNA was in contrast, found in all tissue examined. The levels varied considerably, but were significantly higher in cancers compared to benign tissues ($P<0.05$). Normal human lymphocytes were also found to contain high levels of the 2.5 kb transcript but in 39 malignant breast tumours no association was found between amounts of TGFβ message and the degree of lymphocytic invasion assessed on histological sections. These data suggest a possible autocrine growth role for TGFs in human breast cancer.

**EGF and TGFα found in macrophages present in human carcinomas**

S. Farmer, D.B. Jones, D. Davies & P. Alexander

*CRC Medical Oncology Unit and Department of Pathology, Southampton General Hospital, Southampton SO9 4XY, UK.*

Acid extracts prepared from human and mouse carcinomas were found by ELISA to contain h. and mEGF respectively as well as TGFα. In frozen sections of colonic and mammary carcinomas a proportion of cells stained with a monoclonal antibody (MAB) to h.EGF which does not react with TGFα. Double staining with MABs identifying macrophages (CD11c/3.9) and cytokeratin (5.2) showed unequivocally that in the majority of primary tumours and lymph node metastases EGF is not found in cytokeratin positive cells but is present in some but not all of the cells that stain as macrophages. An MAB to TGEα stained not only all of the epithelial (cytokeratin positive) cells within many of the tumours but also some of the macrophages. The same subpopulation of macrophages appears to contain h.EGF as well as TGFα. This observation is not due to spurious cross reactions as the MAB to TGFα does not recognise h.EGF.

That all macrophages do not contain h.EGF and TGFα is clearly shown in the spleen in which none of the CD11c positive cells bind MABs specific for h.EGF or TGFα. However macrophages present in frozen sections derived from a variety of granulomatous conditions were strongly +ve for h.EGF and TGFα. Freshly separated peripheral blood monocytes stained very weakly or not at all for h.EGF and TGFα but were positive with MAB 3.9. However after culturing for 4 h the adherent monocytes became strongly positive for h.EGF and TGFα. Apparently tissue macrophages stemming from recently arrived monocytes contain h.EGF and TGFα but after a prolonged period of residence these growth factors are lost. These studies suggest that macrophages make a significant contribution to the presence of the EGF class of growth factors within tumours and that there may be considerable overlap between autocrine and paracrine stimulation.

**Effect of anti-EGF antibody on cell growth *in vitro* and tumour growth *in vivo***

J. Blackler, P. Alexander, S. Farmer & D. Davies

*CRC Medical Oncology Unit, Southampton General Hospital, Southampton SO9 4XY, UK.*

Antibodies raised in sheep to mouse EGF were used to test if host derived EGF is required for the progressive growth *in vivo* of low numbers of carcinoma cells which contain TGFα activity but do not make mEGF. ELISA showed that the antibody cross-reacted weakly with hEGF and TGFα but did not inhibit hEGF or TGFα induced mitogenesis. However the IgG inhibited the mintogenic activity of mEGF in a manner that depended both on the dose of IgG and the amount of mEGF used.

*In vivo* anti-tumour activity of the antibody was tested against a transplanted mammary carcinoma which grows from $5\times10^3$ cells i.p. 2.5 mg of IgG was given i.p. three times a week. In the first experiment the mice were killed at 29 days: 5/5 untreated mice had large omental tumours while in the treated group 2/5 were tumour free and 3/5 had small tumours. In the second experiment the mice were followed for up to 3 months: in the untreated group and the group receiving control IgG 8/10 died between 40 and 90 days and 2/10 in each group were alive and tumour free at 100 days. In the group receiving anti-EGF IgG only 3/10 developed a peritoneal tumour but all died from renal failure between days 60 and 93. No anti-tumour activity was observed against sarcoma cells in a similar experiment. The antibody reacted in frozen sections with distal tubules in the kidney. In the serum of mice receiving thrice weekly IgG free antibody circulated at a concentration which inhibited *in vitro* mitogenesis of fibroblasts by 190 ng EGF $ml^{-1}$. The level of mEGF in the urine of mice was not reduced suggesting that urinary EGF does not come from the blood.

### Determination of polypeptide growth factors in the urine of patients with carcinomas

J.W. Sweetenham, D. Davies, J. Blackler & P. Alexander

*CRC Medical Oncology Unit, Southampton General Hospital, Southampton SO9 4XY, UK.*

Urogastrone (hEGF) in urine was measured by a specific two-site ELISA which does not detect mEGF or TGFα. More than 99.99% of the hEGF in urine could be removed by binding to a monoclonal antibody. Absorbed urines were analysed for growth factors other than hEGF by competitive binding to EGF receptors and by mitogenic activity measured by induction of DNA synthesis in density-inhibited foreskin fibroblasts under conditions where EGF, TGFα and PDGF were active but added insulin or transferrin were not. After absorption, urinary hEGF was eluted from antibody and after separation by FPLC (anion-exchange) immune- and receptor-binding activity was found in several fractions, none of which coincided with the peak from plasmid derived hEGF produced in *E. coli.*

The hEGF/creatinine ratio in the urine of untreated patients with colon and breast cancers (and a small number with bladder cancer and hypernephroma) was elevated but within the normal range. The chromatographic profile of hEGF from the urine of cancer patients could not be distinguished from that of normals. Following hEGF absorption, no receptor binding or mitogenic activity could be detected in whole urine.

As synthetic human TGFα is readily separable by FPLC from the hEGF components, urine free of hEGF was separated by FPLC and the mitogenic activity of the fractions measured. PDGF and basic fibroblast growth factor does not bind to the anionic column and would not be detected.

Mitogenic activity was observed in several fractions from cancer patients. Some, but not all of this was blocked by an antibody to EGF receptors which inhibits mitogenicity due to hEGF, mEGF and TGFα.

### Benign thyroid epithelial tumours show escape from IGF-1 dependence for growth

D. Wynford-Thomas, D.W. Williams & E.D. Williams

*Cancer Biology Unit, Department of Pathology, University of Wales College of Medicine, Cardiff, UK.*

We have investigated the proliferative response to growth factors of normal and neoplastic human thyroid epithelial (follicular) cells *in vitro*. Follicles were prepared by collagenase/dispase digestion from histologically normal tissue taken from lobectomies performed for non-neoplastic conditions, and from 6 thyroid adenomas. Follicles were cultured in suspension or as monolayers in serum-free RPMI1640 medium and growth responses assessed by $^3$H-thymidine incorporation and autoradiographic labelling index in successive 24 h periods after addition of growth factor(s).

All 6 batches of normal follicles showed no response to thyrotropin (TSH) or IGF-1 when added singly. However, in the presence of IGF-1 (10 ng $ml^{-1}$) a clear bell-shaped dose-response curve to TSH was seen with a 4–5-fold increase in $^3$H-thymidine incorporation at 0.1 mU $ml^{-1}$. In a representative experiment incorporation at 4–5 d increased from $758 \pm 43$ cpm (mean of 4 cultures ± s.e.) with IGF-1 alone to $3722 \pm 63$ cpm with 0.1 mU $ml^{-1}$ TSH plus IGF-1. The corresponding labelling index rose from 4% to 15%.

In contrast, in 5 out of 6 adenomas a response of equivalent timing and greater than or equal magnitude was obtained with TSH in the absence of any IGF-1. A representative adenoma gave a peak increase in $^3$H incorporation from a basal value of $1050 \pm 70$ cpm to $6998 \pm 600$ cpm with 0.1 mU $ml^{-1}$ TSH alone. The labelling index rose from 9.8% to 33.2%. In the remaining adenoma, a similar labelling index was observed in the absence of any added growth factors.

We conclude that escape from the requirement for exogenous IGF-1 is an early step in the development of human thyroid follicular cell neoplasms. We are currently investigating the possibility that this is due to an autocrine mechanism.

### Multihormonal regulation of prostatic growth responses

T.D. France, M.E.A. Phillips, C.L. Eaton & P. Davies

*Tenovus Institute for Cancer Research, Heath Park, Cardiff CF4 4XX, UK.*

Prostate epithelial cells are considered a paradigm of androgen dependency. However, their growth can be influenced by many other steroid and peptide agents. As adenocarcinoma of the prostate, which arises in epithelial cells, inevitably progresses to androgen-independence these other agents and their mechanisms may be legitimate and more fruitful targets of therapy.

Various prostatic normal and neoplastic cell lines show growth dependence on glucocorticoids, epidermal growth factor (EGF) and insulin. As a corollary, ligand binding assays have detected receptor sites for dexamethasone, EGF and insulin-like growth factor (IGF)-I. Receptors for EGF and IGF-I have also been detected in human prostate specimens. Significant differences in receptor site concentration and affinity have been observed between hypertrophic (BPH) and carcinomatous prostate.

Protooncogene expression in prostate cells has been investigated by quantitative dot (slot)-blot hybridization analysis of RNA. Compared to BPH, c-*myc* expression was elevated in all grades of carcinoma, correlating well with EGF-binding capacity, and c-H-*ras* expression became increasingly elevated with loss of glandular differentiation and appearance of secondary binding sites for IGF-I. Expression of c-*fos* did not change relevant to pathology, but correlated significantly with androgen receptor content. These circumstantial correlations were substantiated using cell lines.

Prostate growth is under the control of several regulatory agents and aberrant growth can be linked to alterations in the capacity to respond and mechanisms of response to these agents.

### Role of protein kinase C activation and c-*fos* gene expression in growth control of a murine macrophage tumour

N.T. Goode & I.R. Hart

*Imperial Cancer Research Fund Laboratories, Lincoln's Inn Fields, London WC2A 3PX, UK.*

Treatment of cells of the murine reticulum cell sarcoma line, M5076, with a range of protein kinase 'C' (PKC) activators had markedly different effects in terms of proliferative response evoked but produced very similar changes in magnitude and kinetics of c-*fos* expression. Thus, the tumour promoters mezerein and the phorbol esters 12-0-tetradecanoyl phorbol-13-acetate (TPA) and phorbol 12,13-dibutyrate (PDBu) [all at 50 ng $ml^{-1}$] exerted a strong anti-proliferative effect, whereas the non-promoting phorbol ester analogue 4α-phorbol 12,13-didecanoate (4αPD) had no effect on this parameter. Analogues of the natural PKC activator diacylglycerol used at 20 μg $ml^{-1}$ did not affect proliferation (1,2-Dioctanoyl Glycerol [DiC8]) or were mitogenic (1-Oleoyl-2-acetyl glycerol [OAG]). However, all PKC activators evoked a rapid and transient increase in c-*fos* gene expression as measured by Northern blotting of poly($A^+$) mRNA with maximum levels of steady state mRNA obtained 15–30 min after initiation of treatment and a decline to resting levels 2 h later.

These results, obtained in a single cell line, show that the induction of c-*fos* gene expression following PKC activation may be a general signal associated with changes in proliferative status but is not deterministic of that change. This hypothesis is in agreement with the observation of induction of c-*fos* gene expression both when fibroblasts and lymphocytes are induced to enter G1 from G0 by mitogenic growth factors and when myelomenocytic cells are differentiated with concommitant cessation of cell proliferation. That divergent proliferative responses may be a consequence of altered expression of PKC isozymes has been investigated using cDNA probes to PKC-α, β and γ on Northern analysis. Similarly, the possibility that changes in cell growth responses may reflect temporal differences in PKC down-regulation has been examined in the M5076 line and the results of these studies will be presented and discussed in the context of regulatory signals and obligatory events in growth control.

### Binding of ligands to the 55 kD component of the interleukin-2 receptor triggers generation of cyclic AMP

A.R. Mire-Sluis, A.V. Hoffbrand & R.G. Wickremasinghe

*Department of Haematology, Royal Free Hospital School of Medicine, Pond Street, London NW3 2QG, UK.*

The receptor for interleukin-2 (IL-2) consists of two non-sulfhydryl linked transmembrane glycoproteins of 55 and 75 kD. Mitogenic activation of T lymphocytes requires the formation of a ternary complex between IL-2 and both the receptor components. We have investigated aspects of the mechanism of transduction of the IL-2 proliferative signal. Binding of IL-2 to its receptor triggers transient generation of cyclic AMP (cAMP). The monoclonal antibody antiTac binds only the 55 kD receptor component, is not mitogenic and can block the mitogenic action of IL-2. AntiTac also stimulates generation of cAMP. When permeabilized lymphocytes were incubated with [$\gamma$-$^{32}$P]ATP both IL-2 and antiTac rapidly stimulated phosphorylation of an 85 kD protein (p85). p85 phosphorylation was also stimulated by addition of cAMP, but not of cGMP or of a protein kinase C activating phorbol ester. We therefore suggest that ligand binding to the 55 kD component of the IL-2 receptor triggers cAMP generation, which activates the cAMP-dependent kinase system, leading to phosphorylation of p85. However, because antiTac is non-mitogenic, other biochemical pathways triggered by ligand binding to the 75 kD receptor component must also be involved in securing commitment of lymphocytes to mitosis.

### Phosphorylation of the calcium/phospholipid-dependent binding protein, p68, in human syncytiotrophoblast plasma membranes

P.D. Webb & P. Kenton

*Department of Immunology, University of Liverpool, PO Box 149, Liverpool, UK.*

Human placental syncytiotrophoblast plasma membrane forms the interface with maternal blood. It is rich in EGF and insulin receptors and expresses the oncotrophoblast antigen placental-type alkaline phosphatase. PLAP is associated with phosphatidylinositol (PtdIns) and can be released from these membranes and tumour cells with exogenous PtdIns-specific phospholipase C (PtdIns-PLC). We have also shown a 'family' of calcium-dependent proteins bind to the trophoblast submembraneous cytoskeleton. These include lipocortins I and II, phosphorylated on tyrosine after EGF treatment, a non-phosphorylated 34 kD protein, probably endonexin, and a 68 kD component not previously shown to be phosphorylated. From Western blots this protein is related to p68, the calcium-dependent binding protein isolated from human lymphocytes. P68 was found to be a major phosphorylated component of trophoblast membranes. It was principally phosphorylated on serine with trace amounts of tyrosine. On incubation of the membranes with EGF there was a time-dependent increase in p68 tyrosine phosphorylation. The phorbol ester, TPA, also stimulated p68 phosphorylation but only at 10 nM or less. At higher concentrations phosphorylation of p68 decreased. Addition of PtdIns-PLC, at concentrations which cleave PLAP from the membranes, also reduced, in a dose-dependent manner, p68 phosphorylation. Since Diacylglycerol generation may accompany this PtdIns-PLC release of PLAP it raises the possibility that the normal mechanism in release of soluble PLAP into the circulation may be associated with modulation of p68 phosphorylation.

### *Ras* oncogene activation in human thyroid tumours

N.R. Lemoine, F.S. Wyllie, V. Wynford-Thomas & D. Wynford-Thomas

*Cancer Biology Unit, Department of Pathology, University of Wales College of Medicine, Cardiff, UK.*

The presence of activated oncogenes in human thyroid cancer was investigated by transfection of genomic tumour DNA into untransformed NIH3T3 mouse fibroblasts. Fibroblast transformation was detected: (a) by the occurrence of dense foci in monolayer, and (b) by the development of tumours in nude mice within 8 weeks after injection (after initial selection for a co-transfected genetic marker).

Five follicular and 11 papillary carcinomas were studied. In only 1 case (a follicular tumour) was transformation detected by the focus assay. In contrast, using the nude mouse assay, all 5 follicular, and 10 of the 11 papillary cases showed transforming activity. No tumours were observed in control transfections using normal mouse or human DNA. DNA from all nude mouse tumours was positive for transforming activity both by focus induction and nude mouse tumorigenesis in second and third rounds of transfection, usually with higher efficiency.

Southern analysis of DNA of transformants derived from follicular cancers identified a human Ha-*ras* gene in 2 cases, N-*ras* in 1 case and Ki-*ras* in 1 case. Oligonucleotide probing demonstrated point mutations in each case involving Ha-*ras* (codon 61 Gln→Arg) and N-*ras* (codon 61 Gln→His), and is in progress for Ki-*ras*. Corresponding analysis for the papillary cancer identified N-*ras* in one and Ki-*ras* in a second case (Ha-*ras* was not identified in any). Oligonucleotide probing demonstrated a point mutation at codon 61, position 2 in the N-*ras* oncogene.

Human thyroid cancer therefore shows a surprisingly high incidence of transforming activity, and in the case of the follicular type, a high incidence of *ras* oncogene activation.

### *Met*-encoded protein phosphorylation in human tumour-derived cell lines

P.R. Tempest & C.S. Cooper

*Institute of Cancer Research, Fulham Road, London SW3 6JB, UK.*

The *met* gene present in the transformed human cell-line MNNG-HOS is activated by a chromosomal translocation in which the 5′ region of the *met* gene (located on chromosome 7) is replaced by promoter sequence derived from an unrelated gene located on chromosome 1. Sequence analysis of cDNA clones indicates that *met* most likely encodes a receptor for an as yet unidentified growth factor. In order to examine proteins encoded by the normal and activated *met* genes we have raised antisera against a synthetic peptide corresponding to the carboxyl terminus of the predicted *met* gene product. The antipeptide antibodies have enabled us to show that the activated *met* gene encodes 60 and 65 kD polypeptides that when incubated *in vitro* in the presence of ATP and $Mn^{2+}$ can catalyse autophosphorylation on tyrosine residues. These studies also provide evidence that the normal unrearranged *met* gene, present in the parental HOS cell line, encodes 140 and 165 kD polypeptides that can catalyse autophosphorylation.

To determine whether activation of *met* can be implicated in the induction of human tumours we have used the *in vitro* protein kinase assay to screen human tumour cell lines for alterations in the product of the *met* gene. Using this assay we have identified altered *met* protein kinase activity in cell lines from two cervical carcinomas, a medulloblastoma and a lung carcinoma. Some of these cell lines possess abnormally high levels of kinase activity of the 140 kDa polypeptide while others exhibit alterations in the size of the *met* gene product. In future studies we hope to discover the significance of these alterations in the product of the *met* gene.

### *Ras* replaces TPA requirement for growth, and induces tumorigenicity, in murine melanocytes

R.E. Wilson, T. Dooley & I.R. Hart

*Imperial Cancer Research Fund Laboratories, Lincoln's Inn Fields, London WC2A 3PX, UK.*

Recently we isolated an immortal line of non-tumorigenic melanocytes requiring the presence of 12-0-tetra-decanoyl phorbol-13-acetate (TPA) for continuous growth (Bennett *et al., Int. J. Cancer,* **39,** 414, 1987). A variant (Mel-ab) of this line has been transfected with various plasmids containing both the aminoglycoside phosphotransferase gene and activated cellular Ha-*ras* or viral Ha-*ras* genes. Transfectants, isolated on the basis of their resistance to G418 ($800\,\mu g\,ml^{-1}$), were examined for *in vitro* growth characteristics and tumorigenic capacity. Untransfected cells, or control transfectants lacking the *ras* gene, only grew in the presence of 150–200 nM TPA. In contrast, cells (Mel-ab pAGT) containing activated cellular Ha-*ras* under transcriptional control of the herpes simplex virus Tk gene grew equally well in the absence or presence of TPA. Moreover, cells containing the viral Ha-*ras* gene under control of a retroviral LTR (Mel-ab LTR *ras*) not only grew well in the absence of TPA but were actually growth inhibited by the addition of TPA (160 nM). The cAMP elevating agent cholera toxin (ct) at $10^{-9}$ M failed on its own to stimulate proliferation but acted synergistically with TPA to induce mitogenesis in untransfected Mel-ab cells. In contrast Mel-ab LTR *ras* cells responded strongly to $10^{-9}$ M ct alone, though this proliferative effect was abrogated completely by TPA.

Tumorigenicity of the various cell lines was monitored by injecting suspensions of viable cells s.c. into thymic, nude mice. Mel-ab cells ($2 \times 10^6$) failed to produce tumours even after 200 days, whereas 5 out of 5 animals injected with $1 \times 10^6$ Mel-ab pAGT or Mel-ab LTR *ras* cells grew >1 cm diameter tumours in less than 20 days. Histological examination and specific stains showed that these tumours were malignant melanomas.

These results show that *ras*-induced tumorigenicity is associated with the induction of autonomous growth.

### A mediating role for topoisomerase II in oestrogenic recruitment of human breast cancer cells

R.J. Epstein[1], J.P. Moore[2], G.I. Evan[2] & P.J. Smith[1]

[1]*MRC Unit, Clinical Oncology and Radiotherapeutics, and* [2]*Ludwig Institute for Cancer Research, MRC Centre, Hills Road, Cambridge, UK.*

Several studies have associated mitogenic activation with a rise in extractable cellular topoisomerase II activity which parallels stimulation of DNA synthesis. Using autoradiography we have shown that oestrogenic stimulation of DNA synthesis in T-47D cells first becomes detectable after 16 h exposure, and that 15–20% more cells are recruited into DNA synthesis by 24 h. Enhancement of drug-induced topoisomerase-II-mediated DNA cleavage by oestrogen is not, however, antagonised by inhibition of DNA synthesis using aphidicolin or hydroxyurea. Stimulation of c-*myc* protein synthesis is maximal within 1–2 h of oestrogen exposure, while elevations in topoisomerase-II-mediated DNA cleavage, cellular topoisomerase II content, and extractable topoisomerase II activity are readily detectable within 4 h of stimulation. No enhancement of topoisomerase-mediated DNA cleavage is seen following inhibition of poly(ADP-ribosyl)ation with 3-aminobenzamide. Flow cytometry confirms that oestrogen stimulation is associated with increased topoisomerase II content in a G1-phase cell subset. These findings suggest (1) that drug-induced topoisomerase-II-mediated DNA cleavage is a useful monitor of enzyme:DNA interaction in intact cells; (2) that oestrogen enhances topoisomerase II synthesis in an activated G1-phase cell subset; and (3) that topoisomerase II may play a controlling, rather than a facilitating, role in chromatin activation.

### Cyclical expression of oestrogen receptor in normal breast epithelial cells does not occur in breast cancer

C. Markopoulos[1,2], U. Berger[2], J.-C. Gazet[1], P. Wilson & R.C. Coombes[1,2]

[1]*Combined Breast Clinic, St George's Hospital, Blackshaw Road, London SW17 0QT, and Ludwig Institute for Cancer Research, St George's Hospital Medical School, Cranmer Terrace, London SW17 0RE, UK.*

We have used a monoclonal antibody (H222) to the oestrogen receptor (ER) to identify receptor in cytological samples obtained by fine needle aspiration (FNA) from women with normal breasts throughout the menstrual cycle. We have also examined biochemically (DCC) ER content of breast cancers in relation to the time in the menstrual cycle during which excision was performed.

In normal premenopausal women ER was detected in the nuclei of epithelial cells in 21/68 (31%) assessable samples, all of which were obtained from women during the first half of their menstrual cycle (days 28 to 14). No sample obtained during the second half of the cycle contained ER.

Analysis of ER content of 83 carcinomas in relation to the menstrual day during which excision was performed showed equal distribution of the ER positive cases throughout the menstrual cycle.

The results of this study on normal breast epithelial cells indicate that ER protein production is suppressed at the time of ovulation in the normal breast epithelium of premenopausal women. In contrast, breast carcinoma cells either synthesise this protein continuously throughout the cycle or fail to express it despite fluctuations of serum hormones.

### Correlation of progesterone receptor immunohistology with radioligand-binding assay and oestrogen receptor immunohistology in breast carcinomas

D. Giri[1], J. Goepel[1], K. Rogers[2] & J. Underwood[1]

*Departments of [1]Pathology and [2]Surgery, University of Sheffield Medical School, Sheffield S10 2RX, UK.*

Radioligand-binding assays for progesterone receptor (PR) suffer disadvantages similar to those described for oestrogen receptor (ER) assays. We therefore evaluated immunostaining by a monoclonal antibody to PR by comparison with radioligand-binding assay for PR as well as ER monoclonal immunohistology on adjacent cryostat sections to minimise sampling differences arising from tumor heterogeneity. Of the 103 samples studied 37 (36%) and 66 (64%) showed significant nuclear staining with the PR and ER monoclonal antibodies respectively. The immunohistological results showed close correlation with the respective biochemical assays ($P<0.0001$). Further the monoclonal antibody study showed that 34 of the 66 cases immunohistologically regarded as ER positive were PR positive, whereas significantly 3 of the 37 deemed as low positive or negative for ER showed strong expression of PR with concordant biochemical assay results. This suggests that in a small number of breast carcinomas constitutive differences may account for PR expression independent of ER. The study clearly shows that nuclear staining by this monoclonal antibody to PR (as previously established for ER (Giri *et al.*, *J. Clin. Pathol.*, **46**, 734, 1987)), correlates strongly with biochemically assayed PR values and appears to be an acceptable alternative to such assays currently favoured in clinical practice. The immunohistological method advantageously identifies, in addition, both the occupied and unoccupied receptor sites and reveals intratumoral cell-to-cell and regional heterogeneity for ER.

### Detection of tumour cells in bone marrow of patients with prostatic carcinoma by immunocytochemical methods

J. Mansi[1,2], U. Berger[1], P. Wilson[1], R. Shearer[2] & R.C. Coombes[1,2]

*[1]Ludwig Institute for Cancer Research, St George's Hospital Medical School, London SW17 0RE, [2]St George's Hospital, London SW17 0QT, UK.*

We have used a cocktail of antisera to prostatic specific acid phosphatase, prostatic specific antigen, epithelial membrane antigen and cytokeratin to examine multiple bone marrow aspirates from pateints with local ($n=15$) and metastatic prostatic carcinoma ($n=15$) and benign prostatic hypertrophy ($n=10$). We found moderate to large numbers of tumour cells in the bone marrow of 11 of 15 (73%) patients with known metastatic disease, and small numbers of abnormal cells in 2 of 15 (13%) patients with apparently local disease. No tumour cells were found in patients with benign prostatic hypertrophy, and only two patients with metastatic disease were found to have tumour cells in their bone marrow when conventional haematomorphological preparations were examined.

These findings suggest that immunocytochemistry can increase the detection rate of metastatic prostatic carcinoma cells. Further follow-up of larger numbers of patients with local carcinoma will reveal whether the presence of micrometastases denotes a poor prognosis.

### The significance of intratumoral aromatase[A] (oestrogen[$E_2$] synthetase) in human breast cancer[BC]

M. Cunha e Silva[1], M.G. Rowlands[2], M. Dowsett[3], U. Berger[1], E.R. Simpson[4], C.R. Mendelson[4], I. Fraytt[5] & R.C. Coombes[1]

*[1]Ludwig Institute for Cancer Research, St George's Hospital Medical School, London SW17 0RE, [2]Institute of Cancer Research, Sutton, Surrey, [3]Chelsea Hospital for Women, London SW3, [4]University Medical School, Dallas, USA, [5]Royal Marsden Hospital, London SW3, UK.*

A proportion of BC depend on $E_2$ for their continued proliferation. Although $E_2$ originates in part from ovarian and adipose tissues, tumour-synthesised $E_2$ by intratumoral A could also be important because of the proximity of this source of $E_2$. In order to determine the significance of intratumoral $E_2$ production we have measured the A content of 114 primary BC removed between 1981 and 1986 using the tritiated water-release assay which measures % conversion of precursors to $E_2$ (interassay variation 5%). 46/114 (40%) % of <0.02% conversion, 48/114 (42%) % conversion of 0.02–0.09% and 20 (18%) showed conversion of >0.09%. There was no relationship between A content and tumour size, histological type, nodal status or ER status. We also examined the relationship between A content and relapse, since 60% of patients have developed overt distant metastatic disease over this period. However, there was no relationship between rate of relapse and A content since 5 yr relapse rate was 65% (<0.02%), 50% (0.2–0.09%) and 45% (>0.09%) ($P=$NS).

In order to localise the enzyme activity more accurately we have utilised monoclonal and polyclonal antisera to A and excellent localisation occurs in ovary and placenta. Some staining is seen in BC tissue but predominantly in stromal cells.

In conclusion, intratumoral $E_2$ production by breast cancer appears unrelated to ER or other prognostic indices and is not a determinant of prognosis. Further studies using immunological reagents are being done to assess the significance of tumour associated A.

### Expression of cytochrome P-450 and glutathione-S-transferase isozymes in human breast tumours

L.M. Forrester[1], J.D. Hayes[2], R.R. Millis[3], L. Bobrow[3], I.S. Fentiman[3], A.H. Harris[4], M. Glancy[1] & C.R. Wolf[1]

*[1]ICRF Laboratory of Molecular Pharmacology and Drug Metabolism, Hugh Robson Building, George Square, Edinburgh, [2]Department of Clinical Chemistry, University of Edinburgh, Edinburgh, [3]ICRF, PO Box 123, Lincoln's Inn Fields, London, [4]Department of Radiotherapy, Newcastle General Hospital, Newcastle, UK.*

The expression of cytochrome P-450 and glutathione-S-transferase (GST) isozymes has been analysed in a number of breast tumour samples. Immunoblotting shows that all breast tumours analysed contained a cytochrome P-450 form associated with the *P-450 C2C* locus. Isozymes within this locus appear to be invovled in steroid hydroxylations which may relate to hormone homeostasis with the tumour and the metabolism of drugs such as tamoxifen. The metabolism of 7-ethoxyresorufin (a marker substrate for *P-450 C2C*) could also be detected in these tumours. Other cytochrome P-450s analysed within the *P-450 C2A, P-450 C2B* and *P-450 C1* loci were not detected in these samples. All breast tumour samples analysed expressed high levels of the acidic ($\lambda$) GST isozyme a protein which has been associated with drug resistance. This could have important implications in their sensitivity to anti-cancer drugs. Variable levels of the neutral ($\mu$) GST isozyme were present in the different tumours. It was intriguing that the basic subunit was only detected in 1 out of 14 samples tested. The activity of glutathione-S-transferase substrates 1-chloro-2,4-dinitrobenzene, ethacrynic acid (acidic) and cumene hydroperoxide (basic) as well as glutathione peroxidase activity have been compared to the GST isozyme content and where possible to the histological sub-type and clinical response to therapy.

**Effect of long-acting somatostatin analogue on the growth of human lung cancer cell lines**

V.M. Macaulay, M.J. Everard, G.P. Joshi, I.E. Smith & J.L. Miller

*Institute of Cancer Research and Lung Unit, Royal Marsden Hospital, Sutton, Surrey, UK.*

Lung cancer cells secrete peptide factors which may contribute to autonomous tumour growth. Bombesin is synthesised by and mitogenic to many small cell lung cancer (SCLC) cell lines (Carney *et al.*, *Cancer Res.*, **47**, 821, 1987). We have shown synthesis of insulin-like growth factor-I (IGF-I) by most SCLC and non-SCLC tissues and cell lines. We examined the effects on human lung cancer cell lines of an inhibitor of peptide secretion. The long-acting octapeptide somatostatin analogue sandostatin was tested on 3 SCLC and 3 non-SCLC lines. The assay system measured 3H-thymidine uptake by cell suspensions in unsupplemented RPMI medium. Two of the 3 SCLC cell lines synthesise and secrete detectable levels of bombesin and IGF-I. However sandostatin 0.001–1000 ng ml$^{-1}$ ($10^{-12}$–$10^{-6}$ M) had no significant effect on DNA synthesis by these lines. None of the 3 non-SCLC lines produce bombesin, but IGF-I is detectable in 2 (NCI-H125, adenocarcinoma; NCI-H226, squamous). NCI-H226 showed inhibition of 3H-thymidine uptake to $68 \pm 6\%$ of control levels ($P < 0.01$) in response to sandostatin 1000 ng ml$^{-1}$. We also saw an effect in one of the 2 adenocarcinoma lines, NCI-H23. There was significant inhibition ($89 \pm 0.5\%$, $P < 0.05$) even at the lowest drug concentration; the effect was maximal at 1000 ng ml$^{-1}$ ($79 \pm 4\%$, $P < 0.01$). In the presence of 10% serum we were unable to show activity in any line. Thus we have demonstrated only modest effects in 2 of 3 non-SCLC but 0 of 3 SCLC cell lines. There was no correlation between response and synthesis/secretion of bombesin or IGF-I. Growth factor secretion may not be a prerequisite for receptor binding and activation. We plan to assess the effect of sandostatin on serial peptide secretion, and to alter drug schedules to maximise growth inhibition.

**Chemotherapy for malignant melanoma: Improved response without survival benefit**

S. Lakhani, P. Selby, J.M. Bliss, M. Cornbleet, M. Mbidde & T.J. McElwain

*Sections of Medicine and Epidemiology, Institute of Cancer Research and Royal Marsden Hospital, Sutton, UK.*

We have carried out a retrospective analysis of 164 patients (pts) with recurrent melanoma seen at the Royal Marsden Hospital between 1976–1986 who had received no previous chemotherapy. They received one of four chemotherapy regimens: Vindesine (VDN) 3 mg m$^{-2}$ weekly (80 pts), melphalan (HDM) 200 mg m$^{-2}$ with autologous bone marrow transplantation (ABMT) (34 pts), BCNU (HDBCNU) 800 mg m$^{-2}$ with ABMT (9 pts) and BOLD regimen (bleomycin, vincristine, CCNU, DTIC) (41 pts). The groups were similar in their age, sex and clinical characteristics. The indications for chemotherapy were progressive disease or uncontrolled symptoms, usually pain. The response to treatment was evaluated using the WHO criteria. The response rates (CR+PR) for the four treatment schedules were: VDN 2.5%, HDM 20.6%, HDBCNU 44.4% and BOLD 24.4%. Although the response rates for HDM, HDBCNU and BOLD were significantly higher ($P < 0.005$) than that for VDN, there was no evidence of prolonged survival with any of the chemotherapy regimens and the choice of treatment was not a significant predictor of survival on multivariate analysis, although responders lived longer than non-responders as usual. Toxicity was predictably greatest with high dose and combination chemotherapies. The results are disappointing. High dose and combination chemotherapy can improve response rates but there are few complete remissions and the increase in partial remissions does not seem to improve survival in this disease. It seems probable that only a treatment which produces *complete* remissions is likely to do this.

**Platelet derived growth factor and insulin-like growth factor II expression in human breast biopsies**

M.T. Travers, P.J. Barrett-Lee, Y. Luqmani & R.C. Coombes

*Ludwig Institute for Cancer Research, St George's Hospital Medical School, Cranmer Terrace, London SW17 0RE, UK.*

The levels of mRNAs for platelet-derived growth factor (PDGF) A and B chains and insulin-like growth factor II (IGF-II) known to be secreted by some human breast cancer cell lines, and to stimulate proliferation of mesenchymal cells *in vitro*, were examined in 51 malignant and 19 non-malignant human breast biopsies by northern and dot blot analysis using human cDNA probes. PDGF-A and B chain transcripts were found in all normal and benign tissue examined. All tumours studied contained PDGF-A transcripts (38/38) and 33/37 (89%) possessed detectable mRNA for the PDGF-B chain. Variations in the levels of A and B chain transcripts were observed in 26 malignant and 8 non-malignant breast samples suggesting a lack of co-ordinated expression of these factors. However, the levels of these transcripts found in lymph node metastases from 5 patients were similar to those found in their respective primary tumours. PDGF-A and B chain expression was also found to be unrelated to oestrogen receptor protein levels. Northern analysis showed a single 4 kb PDGF-B chain transcript and three bands of 2.9, 2.4 and 1.8 kb for PDGF-A. IGF-II transcripts of sizes 6 and 4.8 kb were abundant in all normal and benign tissues (15/15) and were present, but at much lower levels in 11/21 (52%) of carcinomas. The level of stromal invasion was assessed on frozen sections from 30

carcinomas and showed no relationship to the level of A and/or B chain transcripts. The association of IGF-II message with normal and benign tissue may reflect a role for this growth factor in promoting the proliferation of stromal elements. Alternatively stromal cells may themselves secrete this growth factor. Studies involving *in situ* hybridisation of benign and malignant tissue sections may help to resolve these questions.

**PDGF and TGF-B influence proliferation of and extracellular matrix production by mouse embryonic palatal mesenchyme cells *in vitro***

P.M. Sharpe, M.J.M. Carette & M.W.J. Ferguson

*Department of Cell and Structural Biology, University of Manchester, UK.*

Mammalian palatogenesis involves the differentiation of epithelia from 3 regions of the palate (oral, nasal and medial edge), into specific phenotypes. This differentiation is directed by the underlying mesenchyme (Ferguson & Honig, *Curr. Top. Develop. Biol.*, **19**, 137, 1984). Immunocytochemical and proliferative studies on mouse embryonic palatal mesenchyme (MEPM) cells were undertaken to elucidate molecular controls of mesenchyme signalling. Extracellular matrix (ECM) deposition and proliferation were compared when cells were cultured on various substrata (on plastic, on collagen gel and in collagen gel), in response to DMEM/F12 medium supplemented with donor calf serum with or without platelet derived growth factor (PDGF) and transforming growth factor beta (TGF-B). PDGF elicited substrate specific mitogenic responses; stimulation on plastic, no effect on collagen and inhibition within collagen gel matrices. TGF-B exerted inhibition of cell growth irrespective of culture substratum. Immunocytochemical localisation of collagen types I, III, IV, V, IX, fibronectin, laminin, tenascin and heparan sulphate proteoglycan when MEPM cells were cultured on glass or collagen film substrata in the presence of absence of PDGF and TGF-B indicated that with TGF-B present collagen types III, IV, V, fibronectin and laminin were qualitatively more abundant. PDGF had no apparent effect with the exception of an increase in type III collagen production. These results implicate regulatory roles for both PDGF and TGF-B during palate development.

**Differential effects of TGF$\beta$ on normal and neoplastic hepatocytes**

B.I. Carr, R.D. Whitson, T. Fielder, H. Hoshi & K. Itakura

*City of Hope National Medical Center, Duarte, CA 91010, and W. Alton Jones, Cell Science Center, Inc., Lake Placid, NY 12946, USA.*

TGF$\beta$ is a potent non-toxic inhibitor of mitogen-induced DNA synthesis in primary cultures of adult rat hepatocytes (*Cancer Res.*, **46**, 2330, 1986). Since TGF$\beta$ gene expression occurs in rat liver, we investigated whether a variety of human and rat hepatomas differed from normal hepatocytes in response to the inhibitory actions of TGF$\beta$. Hepatocytes or hepatoma cell lines were culture in serum-free medium for 24 h with TGF$\beta$ 0–10 ng ml$^{-1}$. The medium was then changed to fresh medium containing growth factors (hepatocytes) or foetal calf serum (hepatomas) without TGF$\beta$. DNA synthesis was assessed 72–96 h later (normal hepatocytes) or growth curves were measured over the subsequent days (hepatomas). Normal human foetal hepatocytes were similar to rat hepatocytes in the dose responsiveness to the inhibitory actions of TGF$\beta$ with an $ID_{50}$ of 0.5 ng ml$^{-1}$. Growth curves on rat hepatomas H-4-II-E, RH7777 and HTC, and human hepatomas HEP G2, PRC/PRF/5, HEP 3B. HuH-7 and SK-HEP-1 showed no inhibition of growth at any of the tested doses except for HEP 3B and HuH-7 which showed 50% inhibition at 10 ng ml$^{-1}$ TGF$\beta$. The binding of $^{125}$I-TGF$\beta$ was measured on all hepatocyte cell lines. The hepatomas had between 150–800% of the binding of normal rat hepatocytes, measured over 4 h at 4°C. Affinity cross-linking gels showed at least 4 mol wt species of TGF$\beta$ receptor. The hepatoma cell lines produced a factor(s) in their medium which caused a >10-fold increase in DNA synthesis when tested on normal rat hepatocytes and completely antagonized any inhibitory effects of TGF$\beta$. This endogenously-secreted growth stimulant may be important in the resistance of hepatomas to the growth inhibitory action of TGF$\beta$.

**Decreased sensitivity of hepatocytes from regenerating rat liver to the growth inhibitory action of transforming growth factor $\beta$**

A.J. Strain, D.J. Hill & R.D.G. Milner

*Department of Paediatrics, Clinical Sciences Centre, Northern General Hospital, Sheffield S5 7AU, UK.*

Transforming growth factor $\beta$ (TGF$\beta$) is a potent reversible inhibitor of DNA synthesis in adult rat hepatocytes *in vitro*. Our previous work has shown that TGF$\beta$ inhibits DNA synthesis in hepatocytes isolated from normal liver and from regenerating liver 18 h following partial hepatectomy with equal potency (Strain *et al.*, *Biochem. Biophys. Res. Com.*, **45**, 436, 1987). In the present study, the response of hepatocytes from 3 h, 6 h and 12 h regenerating liver was determined. Hepatocytes were isolated by collagenase perfusion and were maintained in serum-free William's E medium for up to 5 days. Medium with or without growth factors was replenished daily and [$^3$H]-thymidine added for the final 24 h of culture.

TGF$\beta$ inhibited DNA synthesis uniformly in hepatocytes from normal liver and from 3 h, 6 h and 12 h regenerating liver in the absence of epidermal growth factor (EGF). However, cells from 3 h and to a lesser extent from 6 h regenerating liver maintained in the presence of 0.85 nM EGF for 72 h were less sensitive to the growth inhibitory action of TGF$\beta$. [$^3$H]-thymidine incorporation was inhibited at 20 pM TGF$\beta$ by only 7% and 33% in hepatocytes from 3 h and 6 h regenerating liver respectively compared with 70% in normal hepatocytes. No change in sensitivity was observed in cells from rats following sham hepatectomy. Within a further 24–48 h, TGF$\beta$ inhibited DNA synthesis by a similar degree in hepatocytes from 3 h regenerating liver in the presence or absence of EGF. These data are compatible with the hypothesis that TGF$\beta$ plays a role as a paracrine growth regulator in adult rat liver. Thus, following partial hepatectomy a transient decrease in sensitivity of hepatocytes to the growth inhibitory action of TGF$\beta$ would result in release of cells from growth restraint.

**The effect of $\alpha$-interferon ($\alpha$-IF) on TGF-$\beta$ cellular mRNA levels in human breast cancer cells *in vitro***

D.J. Kerr[1], A. Sproul[4], S. Cowans[3], W.D. George[3], R.D. Leake[3] & I.B. Pragnell[4]

*Departments of [1]Medical Oncology, [2]Surgery and [3]Biochemistry, Glasgow University, and [4]Beatson Institute for Cancer Research, Glasgow, UK.*

Transforming growth factor-$\beta$ (TGF-$\beta$) has an anti-proliferative effect on epithelial tumour cell lines *in vitro* and may have a role in the regulation of growth of oestrogen receptor positive (ER+ve) breast cancer cells by tamoxifen.

In this study the effect of α-IF (Kirby-Warrick) on the rate of proliferation, phenotypic expression of ER and cellular levels of TGF-β mRNA was determined for ER+ve breast cancer cells (ZR-75 line). Continuous exposure of exponentially growing cells to α-IF (500 IU $ml^{-1}$) significantly increased cell doubling time from 23.2 to 42.5 h ($P<0.05$). Approximately 12 h after initial treatment with α-IF, ER levels had fallen to 15–20% of control values ($P<0.05$) and stayed at this reduced level in the presence of α-IF. RNA was phenol extracted from exponentially growing cells and TGF-β mRNA levels were assessed by standard dot hybridisation with a cDNA probe specific for TGF-β. 24 h following α-IF treatment, TGF-β mRNA had risen by approximately 5–10 fold relative to control and was maintained at this elevated level until α-IF was removed from culture medium. Preliminary studies indicate that for TGF-β has an anti-proliferative effect on ZR-75 cells and it is possible that the cytostatic effect of α-IF is mediated by variation in phenotypic expression of ER and TGF-β.

### The trophic effects of gastrin on the human gastric cell line, MKN45 *in vitro* and *in vivo*

S.A. Watson[1], L.G. Durrant[1] & D.L. Morris[2]

[1]*Cancer Research Campaign Laboratories, University of Nottingham NG7 2RD, and* [2]*Department of Surgery, University Hospital, Nottingham NG7 2UH, UK.*

The recent evidence that the hormone, gastrin, stimulates growth of gastrointestinal tract tumour cells was further investigated.

The established human gastric cell line, MKN45, was evaluated for the presence of gastrin receptors by flow cytometry and found to have a definitive number. MKN45 showed no *in vitro* growth response to gastrin as measured by $^{75}$[Se] selenomethionine incoporation unless thymidine synchronisation of the cell population was induced after which the cells responded trophically (125% of control) to gastrin.

The same cell line was xenotransplanted into nude mice, the resultant tumours were disaggregated and placed in *in vitro* culture where upon synchronisation they showed an even greater trophic response to gastrin (145% of control), but this response was lost on further passaging.

Such xenotransplants were examined for gastrin-dependence *in vivo* and 50–60% of the tumours responded trophically to exogenous gastrin. When the gastrin responsive tumours were retransplanted an overall increase in growth and a greater response to gastrin was achieved with gastrin-treated tumours reaching a median weight of 1.0 g, 7 days before corresponding saline-healed tumours. The overall response to gastrin was further improved by administering the compound continuously via a pump.

It is suggested that gastrin receptor antagonists may have a future role in the inhibition of gastric and colorectal cancer growth.

### Growth inhibition by neurotensin in small cell lung cancer cell lines *in vitro*

J.G. Reeve, J.J. Shaw & N.M. Bleehen

*Clinical Oncology and Radiotherapeutics Unit, MRC Centre, Hills Road, Cambridge, UK.*

Selected human small cell lung carcinoma (SCLC) cell lines contain high levels of neurotensin but fail to express specific cell surface neurotensin binding sites (Goedert *et al., Br. J. Cancer*, **50**, 179, 1984), suggesting that unlike bombesin, this peptide has no autocrine mode of action in these cells. To investigate this possibility, neurotensin was added to SCLC cell lines growing *in vitro* in HITES medium, at concentrations ranging from 50 nM to 5 μM. Cells were counted at intervals during a 7–10 day period. Among the 8 SCLC cell lines (6 'classic'; 2 'variant') neurotensin exhibited dose-dependent anti-proliferative activity. This effect was also seen in a large cell lung carcinoma cell line and in a breast carcinoma cell line. Experiments carried out in RPM1 containing 10% fetal calf serum showed a significant but reduced anti-proliferative effect for neurotensin. Structure-activity studies indicated the importance of COOH-terminal structures as determinants of specific neurotensin binding and biologic action. To date partial $NH_2$ terminal sequences including NT1-10 have no known physiological effects. However, when $NH_2$- and COOH-terminal fragments were assessed for anti-proliferative effects on SCLC cell lines, both were found to be of similar potency as the complete peptide at equimolar doses. These observations, together with the lack of detectable neurotensin binding sites on several of the SCLC cell lines used in this study, indicate that the growth inhibitory action of neurotensin is not mediated via interaction with specific, high affinity neurotensin receptors.

### A potent bombesin antagonist [DArg$^1$, DPhe$^5$, DTrp$^{7,9}$, Leu$^{11}$] substance P, inhibits the growth of human small cell lung carcinoma (SCLC) *in vitro*

P.J. Woll & E. Rozengurt

*Growth Regulation Laboratory, Imperial Cancer Research Fund, London WC2A 3PX, UK.*

Bombesin-like peptides are secreted by many SCLC and may act as autocrine growth factors for these tumours. We have demonstrated that [DArg$^1$, DPhe$^5$, DTrp$^{7,9}$, Leu$^{11}$] substance P (D) is a potent bombesin/gastrin-releasing peptide (GRP) antagonist in mouse fibroblasts (Swiss 3T3 cells) which (i) inhibits DNA synthesis stimulated by GRP; (ii) blocks [$^{125}$I]GRP binding to the GRP receptor; (iii) reduces cross-linking of the Mr 75000-85000 protein putatively a component of the bombesin/GRP receptor; (iv) blocks some of the early cellular events which precede mitogenesis. D also inhibits mitogenesis stimulated by vasopressin, but not that induced by a variety of other growth factors. D is 5-fold more potent than the previously described bombesin antagonist [DArg$^1$, DPro$^5$, DTrp$^{7,9}$, Leu$^{11}$] substance P (A). SCLC cell lines maintained in serum-free medium (RPMI 1640 with hydrocortisone, insulin, transferrin, estradiol, selenium and bovine serum albumin) achieve 10-fold growth in number in about 10 days. The growth of three cell lines was markedly retarded in the presence of A, but could be restored by changing the medium. Both A and D inhibit the growth of SCLC in a concentration-dependent manner, and again D is 5-fold more potent than A. Non-SCLC cell lines are relatively unaffected by D. More potent and specific peptide antagonists of GRP could have therapeutic applications.

### Enhancement of angiogenesis by interleukin-1: Quantitative studies using a rat sponge model

T.-P.D. Fan[1], S.P. Andrade[1] & J. Saklatvala[2]

[1]*Department of Pharmacology, University of Cambridge, Cambridge CB2 2QD, and* [2]*Strangeways Research Laboratory, Cambridge CB1 4RN, UK.*

Tumour angiogenesis is an important control point in the growth of solid tumours. Knowledge about the precise stimulus for such neovascularisation and its mechanism of action will therefore offer clues for immunopharmacological strategies in cancer treatment. Recent studies have shown that polypeptide growth factors such as fibroblast growth factor (FGF) and transforming growth factor (TGF) stimulate the growth of new blood vessels (see Folkman, *NIPS*, **1**, 199, 1986). We report here that interleukin-1 (IL-1) may also be an angiogenic factor. The method (Andrade *et al.*, *Br. J. Exp. Path.*, **68**, 755, 1987) is based on subcutaneous implantation of sterile polyester sponges in rats and subsequent measurement of blood flow in the implants as they become vascularised. Saline, vehicle control or different concentrations of IL-1 in 100 $\mu$l was injected into the sponge via an attached cannula. Histological studies of implants removed at fixed time intervals confirmed that the sponges were gradually infiltrated by host blood vessels. The blood flow in an implant was measured in terms of $^{133}$Xe-saline clearance 6 min after the radioisotope was injected into the sponge via the cannula. Under standard conditions, the $^{133}$Xe clearance from control sponges 16 days post-implantation approached the clearance obtained in normal skin. However, 5 ng of porcine IL-1$\alpha$ (Saklatvala *et al.*, *J. Exp. Med.*, **162**, 1208, 1985) caused accelerated angiogenesis $10 \pm 1$ days post-implantation. Higher doses (20, 50 ng) of IL-1 were also effective, though there were no significant differences between the three test groups. Further work to assess the relative contribution of IL-1, FGF, TGF and other growth factors in this model of angiogenesis is underway.

### Growth factor-induced angiogenesis determined by a new *in vivo* assay

V. Mahadevan[1,2], I.R. Hart[2] & G.P. Lewis

[1]*Department of Pharmacology, Royal College of Surgeons,* [2]*Imperial Cancer Research Fund, Lincoln's Inn Fields, London WC2A 3PX, UK.*

The angiogenic activities of a number of purified growth factors have been determined using a novel *in vivo* assay, first described by Andrade *et al.* (*Br. J. Exp. Path.*, **68**, 755, 1987). A sterile polyether polyurethane sponge disc (1.25 cm x 0.6 cm) with an attached polyethylene cannula (1 cm long) was implanted into the dorsal subcutaneous tissues of groups of young adult Wistar rats. Since clearance of radiolabelled xenon can be used to measure blood flow (Sjersen, *Circ. Res.*, **25**, 215, 1969; Fan & Lewis, *Br. J. Pharmacol.*, **74**, 964, 1981) progressive vascularisation of the sponge was assessed by injecting 10 $\mu$l of Xenon$^{133}$ solution (370 MBq in 3 ml specific activity) into the cannula then at various times monitoring clearance rates using a collimated $\gamma$-detector placed directly over the implanted sponge. Standard clearance curves from controls (PBS only) were compared with those from rats receiving (1) TGF-$\alpha$; 3 $\mu$g on day 7 after implantation, (2) IL-1$\alpha$; 4.8 $\mu$g/day on days 7, 8, 9 and 10 or (3) TNF-$\alpha$; 5 $\mu$g/day on days 7, 8, 9 and 10.

At these doses TGF-$\alpha$ and IL-1$\alpha$ were strongly angiogenic with, for example, nine-minute Xe$^{133}$-clearance values on day 12 post-implantation of 56.8% $\pm$ 1.6 s.e. and 49.5% $\pm$ 0.75 s.e. respectively *versus* 25.4% $\pm$ 1 s.e. for controls ($P \leq 0.001$ Student's *t* test) while TNF-$\alpha$ (27.7% $\pm$ 0.89 s.e.) manifested no angiogenic capacity.

The angiogenesis assay described here is both more humane and reproducibly quantitative than those in current use elsewhere and can be used to determine the angiogenic capacity of any known growth factors.

### Effects of recombinant human interleukin-1 (IL-1) and recombinant human granulocyte-macrophage colony-stimulating factor (GM-CSF) on haemopoietic tissue *in vivo* and *in vitro*

A.L. Jones, B.C. Millar, J.G.B. Bell & J.L. Millar

*Section of Medicine, Institute of Cancer Research, Clifton Avenue, Sutton, Surrey, UK.*

IL-1 is a radioprotector in mice (Neta *et al.*, *J. Immunol.*, **136**, 2483, 1986). We have measured its effect on haemopoietic recovery, as measured by spleen colony forming units (CFU-S), in relation to total body irradiation (TBI) and chemotherapy. IL-1 (2000 U i.p. 20 h before TBI or drug) did not enhance CFU-S recovery after carboplatin, busulphan or melphalan and indeed appeared to exacerbate gut toxicity of melphalan, as measured by crypt survival. However IL-1 at this dose did increase CFU-S post TBI (assessed on day 7 post TBI) between 6 and 14 fold.

Synergistic action with other biologicals may be important. GM-CSF (100 $\mu$g kg$^{-1}$ i.p. bd for 6 days starting 48 h before TBI), increased CFU-S recovery 12 fold. A combination of IL-1 and GM-CSF, administered as above, resulted in an enhanced increase of CFU-S at 26 fold.

*In vitro*, using human bone marrow, the number of granulocyte macrophage colonies (GM-CFUc) increased as a function of GM-CSF concentration up to a maximal value which varied between patients. IL-1 enhanced the response of GM-CFUc to GM-CSF ($P<0.06$): this response was dependent on the dose of IL-1. IL-1 alone did not stimulate GM-CFUc. The best results were seen with GM-CSF 100 ng ml$^{-1}$ and IL-1 320 U ml$^{-1}$.

We conclude that biological agents should be used in combination and may have a role in enhancing haemopoietic recovery after irradiation.

### Preliminary studies of recombinant GM-CSF in man

S. Devereux, D. Campos Costa, J. Gribben, M.F. Spittle, A.M. Jelliffe, A.H. Goldstone & D.C. Linch

*Middlesex and University College Hospitals and the British National Lymphoma Investigation.*

This study has examined the effects of recombinant human granulocyte macrophage colony stimulating factor (rhGM-CSF, Immunex, USA) on haemopoietic progenitor cells and mature phagocytes *in vivo*. In three patients with malignant disease not receiving chemotherapy, GM-CSF given for 10 days caused a modest neutrophilia (230% baseline) and monocytosis (180% baseline). In three patients with resistant Hodgkin's disease treated by very intensive chemotherapy and autologous bone marrow transplantation the average time to reach $1 \times 10^9 \, l^{-1}$ white cells was 13 days and to $0.5 \times 10^9 \, l^{-1}$ neutrophils was 17 days compared to 21 and 23 days respectively in 20 previous controls treated on an identical protocol. This suggests that rhGM-CSF stimulates granulocytic progenitor cells *in vivo* and may be of value in curtailing chemotherapy induced aplasia. Further studies are in progress. rhGM-CSF also has effects on mature phagocytes *in vivo*. One hour infusions of rhGM-CSF in patients with normal blood counts results in rapid transient and profound neutropenia and monocytopenia. Radionuclide studies showed that this was due to transient margination predominantly within the lungs. This *in vivo* phenomenon is paralleled *in vitro* by a rapid calcium dependent increase in expression of neutrophil adhesion proteins and adherence to endothelial cells. In view of this large flux of phagocytes to the lung caution should be exercised in the administration of rhGM-CSF boluses in patients with pulmonary sepsis.

### Phase I/II study of rhG-CSF in patients receiving intensive chemotherapy for small cell lung cancer

M. Hernandez Bronchud[1], T.M. Dexter[2], N. Thatcher[1] & J.H. Scarffe[1]

[1]*Department of Medical Oncology, Christie Hospital and Holt Radium Institute, Manchester, and* [2]*Department of Experimental Haematology, Paterson Laboratories, Manchester M20 9BX, UK.*

Neutropenia as a consequence of chemotherapy is probably the most important factor responsible for increasing susceptibility to infection in patients and hence one of the main reasons for hospital admission during treatment. RhG-CSF is a recombinant protein, similar to the naturally occurring substance which increases the production of neutrophils in man. In our study all patients have the diagnosis of small cell lung cancer and are treated by a combination of adriamycin $50\,mg\,m^{-2}$ i.v. bolus, ifosfamide $5\,gm\,m^{-2}$ + mesna i.v. infusion (both on day 1 of each cycle) and VP16 $120\,mg\,m^{-2}$ i.v. on days 1, 2 and 3. In phase I study (prior to chemotherapy) 7 patients have so far been studied for toxicity and effect on bone marrow (both *in vitro* and *in vivo* parameters) of rhG-CSF administered by continuous i.v. infusion at three different doses (1, 5 and $10\,\mu g\,kg^{-1}\,day^{-1}$). In the phase II study the same patients are given rhG-CSF (as before) on alternate cycles of chemotherapy, acting as their own control. No toxicities were observed during the phase I part of the study – in all 7 patients the number of peripheral neutrophils increased in a dose-dependent manner and granulocyte-function tests proved them to be normally-functioning granulocytes.

During the phase II part of the study the period of neutropenia was considerably reduced while on G-CSF following chemotherapy (with a return to normal or above normal peripheral neutrophil counts within two weeks after day 1).

### Evidence for TNF-α/cachectin production in cancer

F. Balkwill[1], R. Osborne[3], F. Burke[1], S. Naylor[1], D. Talbot[4], H. Durbin[2] & W. Fiers[5]

[1]*Biological Therapy Laboratory,* [2]*Directors Laboratory, ICRF, London WC2A 4PX, ICRF Departments of Medical Oncology,* [3]*St Bartholomews Hospital,* [4]*Homerton Hospital,* [5]*University of Ghent, Belgium.*

We have developed an ELISA assay that detects a labile tumour necrosis factor-α, TNF-α, like activity in freshly obtained serum from cancer patients. 50% of 226 samples from cancer patients with active disease were positive as compared to 3% of 32 samples from normal individuals and 18% of 39 samples from cancer patients with no evidence of disease ($P < 0.0003$). The activity can be absorbed from serum by beads coated with monoclonal antibody to TNF-α. Serum samples from patients with ovarian and oat cell carcinoma were more frequently positive (69% and 63%) than those patients with lymphoma (26% positive). When RNA preparations from peripheral blood mononuclear cells, PBMC, or solid tumours, were probed with TNF-α cDNA, we found evidence for TNF-α mRNA in 8 of 11 samples from cancer patients but only 1 of 8 normal individuals. In addition, TNF-α mRNA was detected in 2 of 6 colorectal tumours. TNF-α may be involved in a host response to cancer, or its production could be part of the process of tumorigenesis. This cytokine could contribute to the symptoms and evolution of some cancers.

### The therapeutic potential of TNF-α and its combination with IFN-γ

F.R. Balkwill[1], D.B. Griffin[1], S. Malik[1] & W. Fiers[2]

[1]*Biological Therapy Laboratory, ICFR, London WC2A 3PX, UK, and* [2]*University of Ghent, Belgium.*

We have studied the antitumour activity of human tumour necrosis factor-α, TNF-α, and human interferon-γ, in human tumour xenograft models. When a range of xenografted cancers (breast, bowel, ovarian) were studied, TNF-α was effective when given locally (intraperitoneally, i.p. to i.p. tumours, intratumourally, i.t. to subcutaneous s.c. tumours) but not systemically. Its combination with IFN-γ was additive or synergistic in the i.p. but not s.c. tumours, resulting in a 3 fold or more increase in lifespan of mice.

The human IFN-γ had no measurable effect on the nude mouse host, but the human TNF-α caused distinctive changes in host cell populations. Within hours of the first injection of TNF-α there was a prominent increase in polymorphs i.p. and in the blood. This declined after several days even with repeated injections but after 14–21 days of daily therapy a pronounced lymphocytosis developed in TNF-α treated mice. Solid i.p. tumours treated with TNF-α showed a marked inflammatory infiltrate and by day 3, central necrosis. Tumour cells became replaced with collagenous material. These changes in solid tumours were more pronounced in the tumour lines that responded to TNF-α with increased mouse survival time.

Financial sponsorship of the meeting was provided by the following commercial organisations: Amersham International; Bioprocessing Limited; Boehringer Corporation (London) Limited; Cambridge BioScience; ICN Biomedicals Limited; Marcel Dekker Publications; Pergamon Journals Limited and John Wiley & Sons Limited.